# Realistic 3D human saccades generated by a 6-DOF biomimetic robotic eye under optimal control

**DOI:** 10.3389/frobt.2024.1393637

**Published:** 2024-05-21

**Authors:** A. John Van Opstal, Reza Javanmard Alitappeh, Akhil John, Alexandre Bernardino

**Affiliations:** ^1^ Section Neurophysics, Donders Center for Neuroscience, Radboud University, Nijmegen, Netherlands; ^2^ University of Science and Technology of Mazandaran, Behshahr, Iran; ^3^ Instituto Superior Técnico, Institute for Systems and Robotics, Lisbon, Portugal

**Keywords:** oculomotor system, main-sequence dynamics, listing’s law, pulse-step control, muscle synergies, component crosscoupling, pulse generation, biomimetic robotic eye

## Abstract

We recently developed a biomimetic robotic eye with six independent tendons, each controlled by their own rotatory motor, and with insertions on the eye ball that faithfully mimic the biomechanics of the human eye. We constructed an accurate physical computational model of this system, and learned to control its nonlinear dynamics by optimising a cost that penalised saccade inaccuracy, movement duration, and total energy expenditure of the motors. To speed up the calculations, the physical simulator was approximated by a recurrent neural network (NARX). We showed that the system can produce realistic eye movements that closely resemble human saccades in all directions: their nonlinear main-sequence dynamics (amplitude-peak eye velocity and duration relationships), cross-coupling of the horizontal and vertical movement components leading to approximately straight saccade trajectories, and the 3D kinematics that restrict 3D eye orientations to a plane (Listing’s law). Interestingly, the control algorithm had organised the motors into appropriate agonist-antagonist muscle pairs, and the motor signals for the eye resembled the well-known pulse-step characteristics that have been reported for monkey motoneuronal activity. We here fully analyse the eye-movement properties produced by the computational model across the entire oculomotor range and the underlying control signals. We argue that our system may shed new light on the neural control signals and their couplings within the final neural pathways of the primate oculomotor system, and that an optimal control principle may account for a wide variety of oculomotor behaviours. The generated data are publicly available at https://data.ru.nl/collections/di/dcn/DSC_626870_0003_600.

## 1 Introduction

**FIGURE 1:**
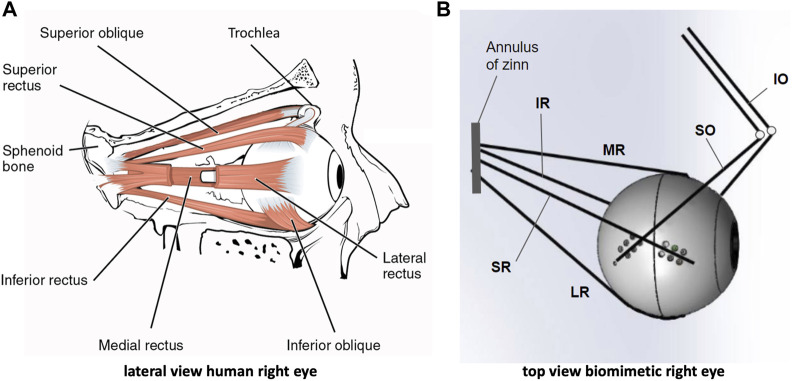
The human eye and its biomimetic robotic equivalent. **(A)** Lateral view of the right human eye showing the insertions of the six extra-ocular muscles. The lateral rectus muscle is partly opened to allow a view of the medial rectus at the nasal side. **(B)** Top view of the biomimetic robotic eye where the muscles are represented by elastic strings. Each string is actuated by its own rotatory motor that rapidly winds the string around a spindle (not shown here, but see Figure 2). The superior and inferior obliques (SO,IO) pull at the eye through pulleys (reminiscent to the SO trochlea of the human eye). The four recti muscles (LR, MR, SR, IR) originate from the annulus of Zinn, which is translated leftward with respect to the center of the eye.

Motion of the human eye is controlled by six extra-ocular muscles that enable the globe to rotate around a fixed center with three degrees of freedom (DOF) through intricate synergistic action: the lateral (LR) and medial rectus (MR) pair induces horizontal rotations of the eye, whereas the superior (SR) and inferior recti (IR), together with the inferior (IO) and superior (SO) oblique muscles, are needed for vertical and cyclo-torsional eye rotations (Figure 1A) (Robinson, 1975; Miller and Robinson, 1984; Hepp and Henn, 1985; Suzuki et al., 1999; Snell and Lemp, 2013).

**FIGURE 2:**
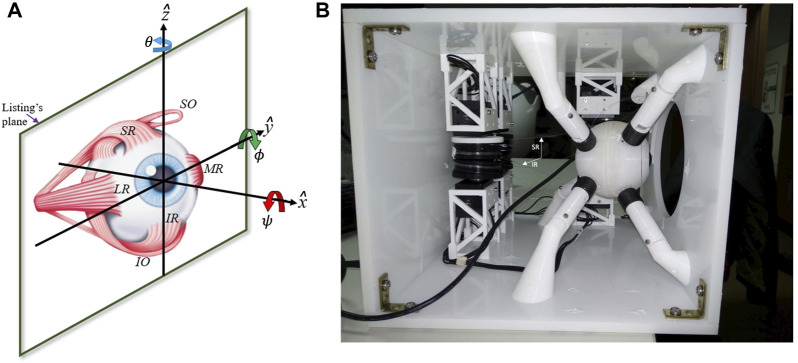
**(A)** The right-handed Cartesian laboratory frame to describe rotation vectors: $\hat{x}$ is the frontal axis for cyclo-torsional rotations (ψ, clockwise positive), $\hat{y}$ the horizontal axis for vertical rotations (ϕ, downward positive), and $\hat{z}$ the vertical axis for horizontal rotations (θ, leftward positive). According to Listing’s Law, rotation vectors that describe 3D eye orientations all lie in Listing’s Plane (tentatively indicated; (11)). **(B)** The physical prototype with its six motors and spindles that control the eye’s 3D orientation by pulling at six thin elastic wires. Four motors are positioned at the back, the other two at the left side of the encasing. The strings representing the SR and IR muscles are identified by the white arrows. The inside of the eyeball contains a camera, with its black cable (‘optic nerve’) leaving the eye at its backside. The eight white supports serve to keep the center of the eye at a fixed location and to provide dynamic friction (after John et al. (2023)).

As the six muscles provide the system in principle with six DOF, measurements of all types of voluntary and involuntary eye movements, like rapid saccades, eye fixations, smooth-pursuit eye tracking, as well as vestibular and optokinetic nystagmus, eye-head coordination, or binocular vergence, have indicated that the instantaneous orientation of the eye only uses two DOF to specify the line of sight at any point in the visual field. Thus, the rotation around the visual axis (cyclo-torsion; Figure 2A) is a task-dependent function of the horizontal and vertical gaze angles: *ψ* = *f*_*task*_ (*θ*, *ϕ*), a property that is known as Donders’ Law (DL; Donders (1870)). Through DL, the oculomotor system would account for the non-commutativity of 3D rotations (Tweed and Vilis, 1987; Tweed et al., 1998). Donders’ Law holds that somehow the redundancy of the oculomotor system regarding its cyclo-torsional state is dealt with by a task-dependent neural control that ties in with the intricacies of the oculomotor plant (Tweed et al. (1998; 1999)). Understanding the underlying mechanisms of how the brain deals with the biomechanics of the eye to control its motions poses an interesting problem for neuroscientists (Robinson, 1975; Miller and Robinson, 1984; Lee et al., 2007), and has also raised considerable controversy in the literature. On the one hand, the emergence of DL has been considered the result of a neural strategy that allows the eye to use the three rotational DOF to control all types of eye movements to optimize both visual and oculomotor function (Tweed and Vilis 1987; Hepp 1990; Van Opstal et al., 1991; Van Opstal et al., 1996; Tweed et al., 1998). In contrast, it has been proposed that the non-commutativity problem is avoided altogether by specific mechanical constraints imposed by the oculomotor plant, e.g., through precisely positioned ‘pulleys’ that guide the muscle trajectories (and hence their effective pulling directions and forces) in an appropriate, eye-orientation dependent, way (Schnabolk and Raphan, 1994; Quaia and Optican, 1998; Demer, 2006; Klier et al., 2006, but see Misslich and Tweed, 2001; Lee et al., 2007). Clearly, the 6DOF neural control and biomechanics of the oculomotor plant form an inseparable duality. Despite the wealth of behavioral measurements of 3D eye- and eye-head movements in human and nonhuman primates, and neural recordings at various levels in the oculomotor system of macaque monkeys, the issue is still not resolved.

Recently, we have adopted a biomimetic approach to study the control of the oculomotor plant (Cardoso, 2019; Dias, 2021; John et al., 2021; Javanmard Alitappeh et al., 2023). We designed a realistic robotic prototype of the human eye with six DOF, which incorporates human-like muscle insertions and properties (Figure 1B). This novel robotic system is driven by six independent rotatory motors that pull at each of the six elastic strings (Figure 2B) to generate a rapid change in eye orientation. To better understand its properties, we derived a detailed computational physical model for this system by applying the Newton-Euler equations for a rotating rigid body (see Supplementary Material).

We investigated how this system could be controlled such that it would generate ocular rotations that resemble human eye movements across the full 3D oculomotor range, in particular, goal-directed saccades. For this type of voluntary eye movement, like for smooth pursuit and steady eye fixations with the head upright and gaze directed at infinity, Donders’ Law reduces the 2D manifold that specifies cyclo-torsion to a plane, which is known as Listing’s Law (LL; Donders (1870); Tweed and Vilis (1987); Van Opstal et al. (1991)).

Several theoretical considerations suggest that saccades may result from a neural speed-accuracy trade-off strategy that aims to direct the fovea as fast and as accurately as possible to a peripheral target (Harris and Wolpert, 2006; Tanaka et al., 2007; Van Beers, 2008; Shadmehr and Mussa-Ivaldi, 2012; Vasilyev, 2019; Varsha et al., 2021). Computational studies on simple models of the oculomotor system with a linear plant have shown that the dynamics of saccades can be understood from such a principle (Harris and Wolpert, 2006; Van Beers, 2008; Shadmehr and Mussa-Ivaldi, 2012). However, whether and how such a strategy also suffices for the full complexity of the 3D eye plant controlled by six muscles, including the emergence of LL and other realistic saccade properties, is not obvious. Although several biomimetic designs of the eye have been described and tested in previous studies (e.g., Peng et al., 2000; Biamino et al., 2005; Beira et al., 2006; Maini et al., 2008; Metta et al., 2010; Oh et al., 2010; Saeb et al., 2011; Schulz et al., 2012; John et al., 2021), none of these works investigated the full dynamics and kinematics of rapid eye movements in 3D with a realistic 6 DOF system.

To drive our 6DOF biomimetic system, we thus implemented an optimal-control algorithm for its physical simulator that minimized a cost function that consisted of the weighted sum of three sub-costs with the different weightings expressing their relative importance: (i) the localization error of the final eye orientation with respect to the goal (any target within the horizontal/vertical oculomotor field); (ii) the total movement duration needed to reach the goal, and (iii) the total energy expenditure of the six motors during the eye-movement trajectory.

In Javanmard Alitappeh et al. (2023) we showed that the system can generate eye-movement trajectories resembling human saccades in 3D with realistic neural control synergies. However, as the simulations were performed on a limited number of saccades, we could not fully report on its eye-movement properties in sufficient detail for lack of statistical rigor. For the present paper, we therefore generated nearly 700 eye movements of the robot’s simulator in three different paradigms and performed a detailed analysis of the movement properties and underlying controls across the 3D oculomotor range. We here quantify the accuracy, trajectories, 3D kinematics, and dynamics of fast goal-directed eye movements, their dependence on movement direction and initial eye orientation, as well as the properties of the underlying motor-control signals (‘neural’ commands) of the six elastic tendons (the extra-ocular ‘muscles’), and compare our results with human and monkey data.

We demonstrate that the resulting movements closely resemble human and monkey saccades that obey the 3D kinematics prescribed by Listing’s Law, the nonlinear main-sequence relations between saccade amplitude and its peak velocity and duration (Bahill et al., 1975; Robinson, 2022), and a nearly complete dynamic synchronization of the motor controls to guarantee nearly-straight saccade trajectories in all directions (Van Gisbergen et al., 1985; Smit and Van Gisbergen, 1990; Van Opstal, 2023). We further show that the muscles become organized in synchronized and appropriate agonist-antagonistic pairs (Sherrington, 1906), and that the ‘neural’ commands resemble the well-known pulse-step control signals that underlie saccade generation at the motor-neuron level in monkey (Fuchs and Luschei, 1970; Fuchs and Luschei, 1971; Robinson and Keller (1972); Suzuki et al. (1999)).

## 2 Methods

### 2.1 The eye model and the nonlinear simulator

Table 1 provides the hierarchical nomenclature used to address the different hardware and software components of our biomimetic robotic system.

**TABLE 1:** The hierarchical nomenclature of our biomimetic robotic system.

	Nomenclature
*Prototype*	the hardware implementatation of the biomimetic eye
*Model*	the set of physical equations that describe the prototype
*Simulator*	the numerical (Matlab) implementation of the model
*Approximator*	the NARX neural network approximation of the simulator

#### 2.1.1 The model

Similar to the human eye, the robotic eye rotates around its fixed center as soon as the six elastic tendons apply a net torque. The tendons are affixed to the globe at contact points that enable rotational movements with three degrees of freedom (see Figures 1B; Figure 2B). These contact points were determined and appropriately scaled from measurements of the human eye (Miller and Robinson (1984); Supplementary Material). Employing a dedicated rotatory motor for each tendon that pulls the tendon around its spindle allows for a fast control of the eyeball in six directions, which approximate left-right, up-down, and cyclo-torsional rotations in clockwise and counterclockwise directions. The Newton-Euler equations describing the dynamics of the oculomotor plant result to be highly nonlinear, which is due to several factors: (i) to changes in the cable pulling directions as function of the 3D orientation of the eye, (ii) to the associated eye-orientation dependent changes in the moment of inertia of the globe, and (iii) to the inherent limitation that muscles can only exert pulling forces, and not push. Further, (iv) the relationship between the 3D orientation of the eye, its angular velocity, and its rate-of-change of orientation is nonlinear because it includes the vector product (below, (3). The quantitative details of the underlying equations and their computational implementation are provided in Javanmard Alitappeh et al. (2023) and in the Supplementary Material.

#### 2.1.2 The optimal control algorithm

In the Optimal Control of the nonlinear simulator, we included three sub-costs that jointly served to minimize the total movement cost. Finding the optimal control for a given saccade involves two computational loops (Shadmehr and Mussa-Ivaldi, 2012): in the first loop, the total movement cost is optimized for saccades of different durations, *D*, between 30 and 210 ms, which we sampled in relatively coarse steps of 20 ms. The second loop finds the duration for which the total cost reached a minimum (Eq. 1). The three costs were.

(i) The *accuracy* cost (*J*_*A*_(*D*); quadratic) quantifies the squared error of the movement endpoint at time *D* with respect to the target goal. The larger the error, the higher the cost.

(ii) The *duration* cost (*J*_*D*_(*D*); hyperbolic, Shadmehr et al. (2010)) expresses the desire that the time needed to reach the goal (‘reward discount’) should be as short as possible.

(iii) The *energy* cost (*J*_*E*_(*D*); quadratic) quantifies the total kinetic energy consumed by the six motors during the trajectory for time *t* ∈ [0, *D*].

The optimal saccade is the one for which the duration has the lowest total cost, calculated as

$$D_{\mathrm{OPT}}=\underset{D}{\mathrm{arg min}}\left[J_{\mathrm{MOV}}\left(D\right)\right]=\underset{D}{\mathrm{arg min}}\left[\lambda_{A}J_{A}\left(D\right)+\lambda_{D}J_{D}\left(D\right)+\lambda_{E}J_{E}\left(D\right)\right]$$ (1)

where the three weighting factors, *λ*_*α*_, *α* ∈ {*A*, *D*, *E*}, were obtained by trial and error, to ensure a convex *J*_*MOV*_(*D*) function^1^ with a clear minimum. Details of the algorithm are given in Javanmard Alitappeh et al. (2023) and summarized in the Supplementary Material.

#### 2.1.3 Neural-network (NARX) approximation

Finding the optimal controls for the nonlinear Newton-Euler equations from the discretized simulator of the robotic prototype is computationally hard, as it requires tedious calculations of local derivatives that need to be redone for every change made to the prototype. Further, these derivatives only provide accurate local first-order approximations for a few degrees around the evaluation point (Dias, 2021). To significantly speed up and generalize this procedure, we instead used an alternative approach with a recurrent neural network (a Nonlinear Autoregressive Network with Exogenous inputs, or NARX Thuruthel et al. (2017)). The NARX architecture acts as a general, model-free, and flexible approximator, than can be readily trained on any complex nonlinear system.

**FIGURE 3:**
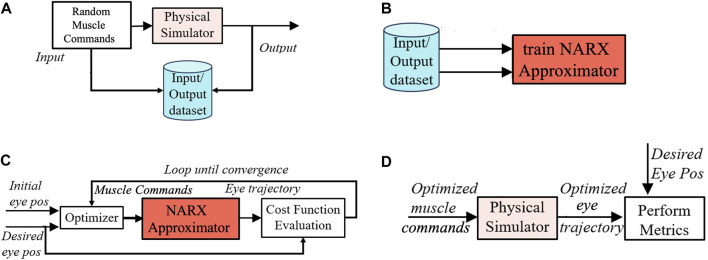
The control algorithm for the simulator of the physics model of the robotic prototype. **(A)** First, a long series of pseudo-random binary step-control signals (PRBS) to the muscles is passed through the physics simulator to generate a large input-output data base. **(B)** Subsequently, this data base is used to train the NARX as an accurate approximator for the nonlinear physics simulator. **(C)** After training, the NARX approximator is used to optimize eye-movement trajectories by minimizing the costs of the optimal control for each target-saccade pair. **(D)** Finally, the optimized controls are used to drive the physics simulator, from which the various eye-movement properties are extracted. Taken together, this procedure is far more efficient than optimizing the trajectories directly from the physical simulator.

To train the network, we obtained an extensive input-output data set, sampled at every 1 ms, with a total length of 2 ⋅ 10^6^ ms. Inputs to the muscles were generated as a pseudo-random binary step sequence (PRBS) that was passed through the simulator. PBRS signals are useful for systems identification because they have a white spectrum and cover a broad workspace. The NARX network was trained until it approximated the input-output sequence of the simulator with sufficient accuracy (see Figure 3A; Javanmard Alitappeh et al. (2023)). Once the NARX approximator was trained, the optimal controls for the tendons were found by using the NARX as an accurate and flexible approximator for the nonlinear physical simulator (Figure 3B).

#### 2.1.4 Numerical implementation

Figure 3 illustrates the different steps involved in the numerical implementation of our algorithms for the robot’s simulator. By specifying the *initial* and *desired* final eye orientations, (as shown in Figure 3C), the optimal control algorithm included several computational modules: An *i*) *optimizer function* that searches for the best set of motor commands to move the eye from the initial to the final desired gaze direction. *ii*) *A cost-evaluation module* that assesses the quality (cost) of candidate motor commands by calculating and adding the three sub-costs. The cost will depend on the trajectory of the eye movement, which is found by simulating the *iii*) *NARX approximator module*. After finding the optimized muscle commands for a specific goal with the NARX through^1^, we utilized the actual physical simulator to generate the model’s eye movements. We subsequently evaluated the performance of the system by analyzing the different properties of a resulting set of eye movements (See Figure 3D), as described in the next sections.

In the present Matlab implementation of the simulator and NARX approximator (The Mathworks, version 2022b), running on a MacBook Pro (2019) with a 2.3 GHz, 8-Core Intel Core i9 processor and 32 Gb RAM, finding the optimal controls for a single saccade took approximately 180 s.

### 2.2 Simulations

We generated three eye-movement data sets with the trained and optimized physical simulator: (1) The *Zero-Initial Paradigm* generated a data set of 199 saccades, where every saccade started from [0,0,0], and the 2D goals were drawn at random from the range within [*G*_*y*_, *G*_*z*_] ∈ [−0.3, + 0.3] rad/2 and *G*_*x*_ = 0. (2) The *Continuous Paradigm* yielded a data set of 298 saccades, where again the 2D targets were drawn at random within the same range as in the zero-initial paradigm, but now each saccade started where the previous saccade ended. In this way, the saccade amplitudes ranged between [0, 50] deg, starting from a wide range of initial eye orientations. (3) A *Horizontal Continuous Paradigm* elicited 202 purely horizontal saccades where all target locations were drawn at random on the horizontal axis from *G*_*z*_ ∈ [−0.3, + 0.3] rad/2 in 0.012 rad/2 intervals, keeping [*G*_*x*_, *G*_*y*_] at zero (for data access, see Data Availability Statement).

**FIGURE 4:**
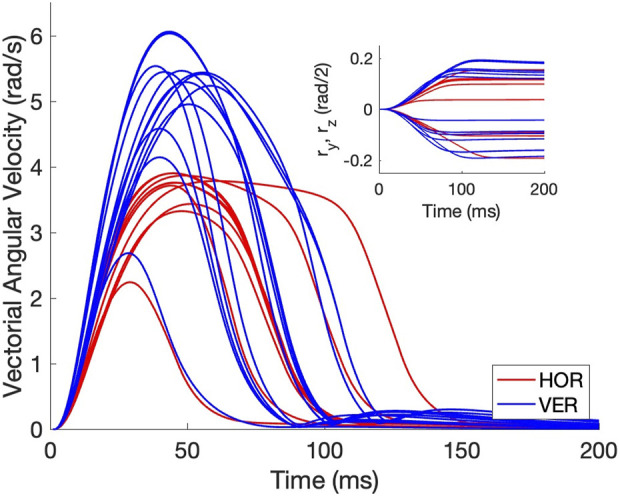
Example vectorial velocity profiles (in rad/s) and corresponding eye-position traces (inset shows the associated rotation-vector components) of purely horizontal (red) and vertical (blue) eye movements from the zero-initial paradigm. Note that the movements do not overshoot and that vertical movements reach higher peak velocities than the horizontal movements. Furthermore, all profiles are single-peaked.

In Figure 4 we provide some illustrative examples of the eye-movement dynamics of the simulated biomimetic eye. We here selected vectorial velocity profiles from 10 purely horizontal (red) and 13 purely vertical (blue) eye movements from the zero-initial paradigm, with their corresponding eye-position traces (inset). In the analyses that follow, we extracted a set of parameters from these profiles such as the peak velocity of the vector, but also of its components, the eye-movement duration, the curvature of the spatial trajectory, as well as the properties of the motor-control signals that underlie these dynamics.

In the Results, we primarily show the data from the *Continuous Paradigm* across the oculomotor range. The data from the *Zero-Initial Paradigm* resulted to be very similar and are provided as Supplementary Material. The data from the Horizontal Continuous Paradigm served to generate Figure 12.

### 2.3 Data analysis

The state of the eye (Eq. 2) is described by its 3D orientation in a right-handed, head-fixed Cartesian coordinate system [*x*, *y*, *z*] (Figure 1A), in which *x* = frontal axis (ocular cyclotorsion, clockwise positive), *y* = horizontal axis (vertical eye orientations, downward positive), *z* = vertical axis (horizontal eye orientations, left positive) in Euler-Rodrigues rotation-vector notation, and the associated 3D angular velocity vector (Eq. 3):

$$x\left(t\right)\equiv \left[r_{x}\left(t\right),r_{y}\left(t\right),r_{z}\left(t\right),\omega_{x}\left(t\right),\omega_{y}\left(t\right),\omega_{z}\left(t\right)\right]=\left[r\left(t\right),\omega \left(t\right)\right]$$ (2)

where

$$\omega \left(t\right)=\frac{2\left(\dot{r}\left(t\right)+r\left(t\right)\times \dot{r}\left(t\right)\right)}{1+\|r\left(t\right){\|}^{2}}$$ (3)

with $\dot{r}\equiv dr/dt$ (coordinate velocity, or the rate of change of orientation) and × is the vector cross product (Hepp, 1990; Hess et al., 1992; Haslwanter, 1995; Van Opstal, 2002).

Time *t* ∈ [0, *D*] is sampled in 1 ms time steps, with *D* the saccade duration, which was discretized in 20 ms intervals in the optimal control algorithm (see above). The goal for the eye (Eq. 4) was specified by a stationary target state, expressed in the laboratory frame:

$$x_{G}\left(t\right)\equiv \left[r_{G},\omega_{G}\right]=\left[0,G_{y},G_{z},0,0,0\right]$$ (4)

The goal served directly as target for the zero-initial paradigm, but for the continuous paradigms it was re-calculated as the rotation, ***q***_*ROT*_, that brings the eye from the initial position, ***r***_*on*_ (i.e., the final position of the previous eye movement), to the goal, ***r***_*G*_. It was calculated by (Hepp, 1990):

$$q_{\mathrm{ROT}}\equiv r_{G}◦r_{on}^{-1}=\frac{r_{G}-r_{on}+r_{on}\times r_{G}}{1-r_{on}•r_{G}}$$ (5)

with ◦ the rotation-vector product and • the vector dot-product. Note that from Eq. 5 ***q***_*ROT*_◦ ***r***_*on*_ = ***r***_*G*_.

We quantified eye-movement accuracy by determining least-squared error linear regression lines (Eq. 6) for the horizontal (azimuth) and vertical (elevation) angles of the final eye orientation vs. the target location:

$$\begin{array}{cc} \theta_{H} & =a+b\cdot T_{H}\\ \phi_{V} & =c+d\cdot T_{V} \end{array}$$ (6)

with azimuth, *θ*_*H*_ ≡ − 2 arctan (*r*_*z*_), elevation, *ϕ*_*V*_ ≡ − 2 arctan (*r*_*y*_), and the associated target angles *T*_*H*,*V*_ ≡ − 2 arctan (*G*_*z*,*y*_), all in deg (where we adopt the convention that rightward and upward angles are taken positive). [*a*, *b*, *c*, *d*] are the regression parameters found by minimizing the mean squared error with Matlab’s *regstats* routine. The quality of the fit was specified by the coefficient of determination, *r* ^2^, which indicates the variability in the data explained by the regression (with *r* Pearson’s linear correlation coefficient).

To investigate the main-sequence dynamics of the eye movements, we fitted the following two affine relations (Bahill et al., 1975; Van Opstal and Van Gisbergen, 1987):

$$\begin{array}{cc} D & =e+f\cdot R\\ V_{PK}\cdot D & =k+m\cdot R \end{array}$$ (7)

where the saccade vector in Eq. 7 is determined by its amplitude $R=\sqrt{\theta_{H}^{2}+\phi_{V}^{2}}$ , and its direction Φ = arctan (*ϕ*_*V*_/*θ*_*H*_), both in deg. Combination of these relations predicts for the peak eye velocity the following relationship with amplitude:

$$V_{PK}=\frac{k+m\cdot R}{e+f\cdot R}\approx \frac{m/e}{1/R+f/e}$$ (8)

Note that Eq. 8 saturates for large amplitudes at *m*/*f* deg/s; in the right-hand side we ignored the small offset *k*, thus assuring that *V*_*PK*_ = 0 for *R* = 0.

To characterize the saccade trajectories, we estimated their curvature by the normalized maximum distance of eye orientation from the line connecting the start- and end orientations (Smit and Van Gisbergen, 1990). To that end, we first translated all saccade trajectories in the horizontal-vertical plane to the origin by subtracting the initial eye orientation, [*θ*_*H*_ (0), *ϕ*_*V*_ (0)], and then rotated all translated saccade trajectories towards the horizontal axis with

$$\begin{array}{cc} \theta_{H}^{\mathrm{rot}}\left(t\right) & =\;\;\cos\Phi \cdot \theta_{H_{TR}}\left(t\right)+\sin\Phi \cdot \phi_{V_{TR}}\left(t\right)\\ \phi_{V}^{\mathrm{rot}}\left(t\right) & =-\sin\Phi \cdot \theta_{H_{TR}}\left(t\right)+\cos\Phi \cdot \phi_{V_{TR}}\left(t\right) \end{array}$$ (9)

We subsequently determined the maximum absolute vertical deviation of the rotated trajectory of Eq. 8, ${\left[\theta_{H}^{\mathrm{rot}}\left(t\right),\phi_{V}^{\mathrm{rot}}\left(t\right)\right]}^{T}$ , normalized by the saccade amplitude, and distinguishing clockwise (positive) vs. anticlockwise (negative) curvatures by Eq. 10 as in Smit and Van Gisbergen (1990), by:

$$C\equiv -\text{sign}\left(\Delta \theta_{H}^{\mathrm{rot}}\right)\cdot \frac{\text{max}\|\phi_{V}^{\mathrm{rot}}\left(t\right)\|}{\left\|\Delta \theta_{H}^{\mathrm{rot}}\right\|}$$ (10)

In this way, a rightward semicircular trajectory with its arc in the first quadrant yields *C* = −0.5. We considered trajectories to be straight when |*C*| < 0.03 and really curved for |*C*| > 0.15.

We determined Listing’s plane (LP) according to Eq. 11 by fitting the following relation through the instantaneous 3D eye-orientation data, expressed as Euler-Rodrigues rotation vectors (Van Opstal et al. (1991); Hess et al. (1992); Figure 2A),

$$r\left(t\right)={\left[r_{x}\left(t\right),r_{y}\left(t\right),r_{z}\left(t\right)\right]}^{T}\;\;\text{with LP:}\;\;r_{x}\left(t\right)=\alpha \cdot r_{y}\left(t\right)+\beta \cdot r_{z}\left(t\right)$$ (11)

According to the so-called *common-source model* of the saccadic system (e.g., Van Gisbergen et al., 1985; Smit et al., 1990; Van Opstal, 2023), oblique saccades are generated by a central nonlinear vectorial pulse generator, causing the horizontal and vertical velocity commands to be scaled versions of each other through linear vector decomposition of the velocity command (Eq. 12). This simple model predicts that, as a consequence of the nonlinear main sequence (Eq. 8), the peak velocity of a component, i.e., either Δ*H* or Δ*V*, should vary with the direction of the saccade vector, according to:

$$V_{PK}\left(\Delta H,\Phi \right)=\frac{\left(m/e\right)\cdot \cos\Phi }{1/\Delta H+f/e}\;\;\;\;\text{and}\;\;\;\;V_{PK}\left(\Delta V,\Phi \right)=\frac{\left(m/e\right)\cdot \sin\Phi }{1/\Delta V+f/e}$$ (12)

where we here simply assume a single main-sequence relation for all saccade directions (i.e., *m*, *e* and *f* are not Φ-dependent). Note that Eq. 12 directly follows from Eq. 7 and has no independent free parameters. For a fixed component amplitude, the peak velocity is then predicted to vary according to *V*_*PK*_(Δ*H*, Φ) = *V*_*Pk*_(Δ*H*, 0) ⋅ cos Φ, and *V*_*PK*_(Δ*V*, Φ) = *V*_*Pk*_(Δ*V*, 0) ⋅ sin Φ, respectively.

In the Results, we explore and quantify these behavioral relationships for the eye movements generated by our biomimetic eye simulator.

## 3 Results

### 3.1 Eye-movement accuracy

**FIGURE 5:**
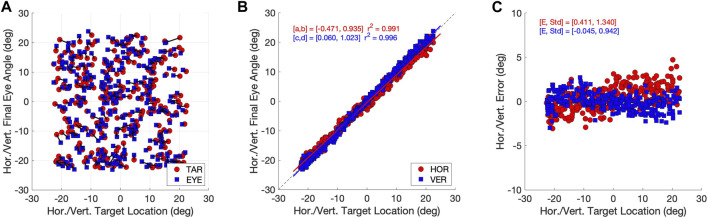
Accuracy of the model eye movements (continuous paradigm). **(A)** Target locations (red dots) and eye-movement end points (blue squares) as azimuth/elevation angles in deg. Associated stimulus-response pairs are connected by solid lines. Note that most saccade end points fall close to the target location. **(B)** Stimulus-response relations for the horizontal (red) and vertical (blue) saccade vector components. Both relations are well described by linear regression lines with a slope close to one and an offset near zero deg. The coefficient of determination, r^2^ > 0.99. **(C)** Signed localization errors from the data in **(A)** (in deg) as function of horizontal (red) and vertical (blue) target angles. The mean errors are close to zero deg, with a standard deviation of around one deg.

In Figure 5 we quantify the accuracy of the responses from the Continuous eye-movement paradigm. The accuracy of the eye movements was high. Figure 5A shows the eye-movement end points (blue), connected to the associated target locations (red), indicating that the localization errors were typically small. In Figure 5B we quantified the accuracy of the horizontal and vertical gaze directions by linear regression (Eq. 6) of the data in Figure 5A. Both regression lines have slopes close to 1.0 and offsets close to zero deg, while the coefficient of determination was very close to 1.0, indicating little variability. In Figure 5C we show the errors of each eye movement, on an expanded scale. The standard deviations of the errors for the two components were close to one deg. A similar sacade accuracys is reported for saccades made by humans, monkeys, or cats (Robinson, 2022). The results for the Zero-Initial Paradigm were similar (Supplementary Material).

### 3.2 3D eye-movement kinematics

**FIGURE 6:**
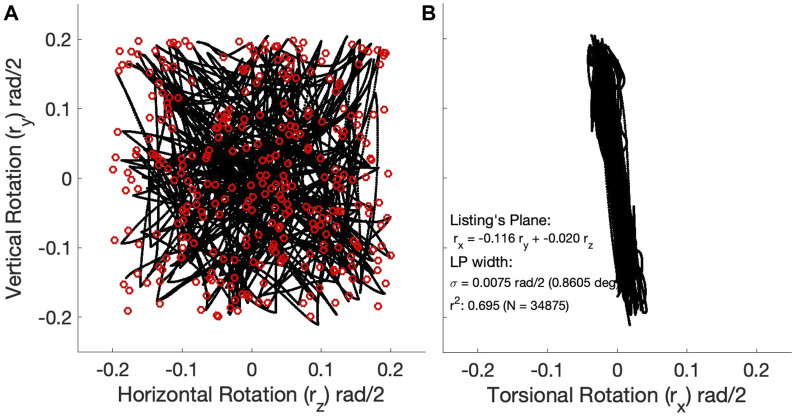
3D kinematics of the 298 eye-movement trajectories (almost 35,000 data points) generated by the continuous paradigm. **(A)** Trajectories in the horizontal (r_z_) - vertical (r_y_) plane were expressed as rotation-vector components in half-radians. Red open symbols represent the target locations. **(B)** The same trajectories seen in the torsional (r_x_) - vertical (r_y_) plane. Note that the 3D trajectories are confined to a 2D manifold, well-described by a plane, Listing’s Plane (r = 0.83), with a width (std) of less than one deg. The plane is slightly tilted (leftward rotation around the z-axis, which sticks out of the image plane) by about 6.6 deg (θ_LP_ = − arctan (0.116)).

Figures 6A,B shows two planar projections of the 3D kinematics of the instantaneous eye orientations (expressed as Euler-Rodrigues rotation vector components) for the eye movements of the Continuous Paradigm (N = 34,875 data points). Note that the data are expressed in the laboratory frame where **r**=(0,0,0) is the straight-ahead orientation of the eye. The *xy*-projection in Figure 6B shows that the eye-orientation data are confined to a plane (Eq. 11), which is well described by

$$r_{x}=-0.116\cdot r_{y}-0.020\cdot r_{z}\;\;\;\left(r^{2}=0.695\right)$$ (13)

The width of the best-fitted plane in Eq. 13 is *σ* = 0.075 rad/2, which corresponds to 0.86 deg. This precision is quite similar to that reported for monkey eye movements (e.g., Hess et al., 1992). As the plane is slightly tilted leftward in the *xy* projection, a horizontal rightward rotation of 6.6 deg aligns the data with Listing’s frame of reference (Eq. 14), where ***r*** ^***L***^ = [0,0,0] refers to the physiologically defined *primary position* (i.e., 6.6 deg to the right of straight ahead in the laboratory frame), and Listing’s Law simply reads

$$r_{x}^{L}=0$$ (14)

The observed tilt of Listing’s Plane within the laboratory frame is due to mechanical asymmetries in the pulling directions of the muscular system with respect to the frame’s origin (Haustein, 1989; John et al., 2021). This phenomenon is also observed in human and monkey data (Hess et al., 1992; Van Opstal et al., 1996). Very similar results were obtained for the Zero-Initial Paradigm (Supplementary Material.

### 3.3 Eye-movement dynamics

**FIGURE 7:**
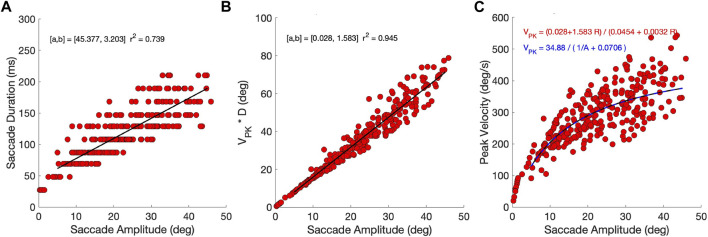
Main-sequence dynamics (continuous paradigm). **(A)** The models’ eye movements follow the main-sequence relations of saccades. Saccade duration has an affine relation with movement amplitude. Note that durations were sampled at 20 ms intervals in the optimal control algorithm (see Methods). **(B)** The product of peak eye velocity with saccade duration is strongly related to saccade amplitude, in line with single-peaked velocity profiles. **(C)** The relations in **(A)** and **(B)** predict that the saccade peak eye velocity saturates at large saccade amplitudes at 34.88/0.0706 = 1.583/0.0032 = 494 deg/s. The predictions are shown as solid black and blue-dashed lines. Note that the scatter around the predictions is due to the fact that saccade velocities vary with the saccade direction and initial eye orientation (see Figures 11, 12).

To check whether the model’s responses indeed resembled saccades, we quantified the main-sequence relationships for the eye-movement data (Bahill et al., 1975). Figure 7A shows that, despite the relatively coarse sampling of tested movement durations at 20 ms intervals (see Methods, Optimal Control), the optimal movement duration increased in a nearly affine way with the eye-movement amplitude. As the saccade velocity profiles are predominantly single-peaked (e.g., Figure 4), they are expected to obey a tight linear relation between the saccade amplitude and the product of its peak velocity and duration (in deg), with a slope that ranges between 1.5 and 1.8 (Van Opstal and Van Gisbergen, 1987). Figure 7B shows that this was indeed the case for the biomimetic eye movements. The offset (0.028 deg) is practically zero, and the slope of 1.583 is close to that reported for human data. The linear relation explains >94% of the variability in the data. This indicates that the biomimetic eye movements are indeed reminiscent to human and monkey saccades. Combining the results of panels (A) and (B) then leads to the prediction that the peak velocity of the saccades (Eq. 8) should vary with amplitude according to

$$V_{PK}=\frac{34.88}{1/R+0.0706}\;\;\text{deg/s}$$ (15)

which saturates at 494 deg/s for *R* → *∞*. This relation is shown by the dashed blue line in Figure 7C and Eq. 15 is in line with reported human data (Bahill et al., 1975; Robinson, 2022). The relatively wide scatter of the data around the predicted line is due to the fact that the saccade peak velocity does not only depend on amplitude, but also on the saccade direction and the initial eye orientation (further analysed below). The simple equation therefore only describes the average behavior of the saccades across all initial conditions and directions. The Supplementary Material provides the results for the zero-initial paradigm, in which the variability due to the changes in initial orientation is absent.

### 3.4 Curvature of trajectories

**FIGURE 8:**
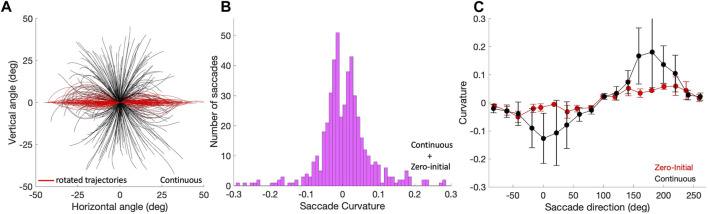
Saccade curvature. **(A)** Saccade trajectories of the continuous paradigm were translated to the origin (black traces), and subsequently rotated by their overall vector angle towards the horizontal axis (see Methods; red traces). Curvature is then calculated by applying Eq. 10 to the rotated trajectories. **(B)** The distribution of curvature for the 498 trajectories of the pooled zero-initial and continuous paradigms peaks strongly around zero, indicating that the far majority of saccade trajectories is approximately straight. **(C)** Curvature varies in a systematic way with the saccade direction, here shown separately for the zero-initial (red) and continuous (black) paradigms. Curvature and its variability are higher for the latter saccade population. Means (solid dots) and standard deviations were calculated over the data points falling in a sliding window of 20 deg wide and 10 deg overlap.

To assess whether the saccade trajectories were straight, we quantified their curvature by applying Eq. 10. Figure 8A shows the applied procedure in Eq. 9 and the result of this analysis as a histogram in Figure 8B for the pooled Zero-Initial and Continuous sets of nearly 500 saccades. A large number of saccade trajectories (239/498; 48%) had |*C*| < 0.03 and could be qualified as virtually straight. Only a small minority of 39/498 (∼7%) of the trajectories had |*C*| > 0.15, and were therefore characterized as substantially curved. The obtained curvature values fall well within the range reported for human saccades (Smit and Van Gisbergen, 1990). None of the 199 zero-initial saccades belonged to the latter category, as can be seen in Figure 8C (red). Saccades from the continuous paradigm were more variable in their curvature, especially for those with near-horizontal directions (Figures 8A,C, black traces).

Straight oblique saccade trajectories entail that the profiles of their horizontal and vertical velocity components should be highly synchronized and scaled versions of each other: ${\dot{\theta }}_{H}\left(t\right)\propto {\dot{\phi }}_{V}\left(t\right),\;\forall t$ . To verify that this was indeed the case, we correlated ${\dot{\theta }}_{H}\left(t\right)$ vs. ${\dot{\phi }}_{V}\left(t\right)$ for all oblique saccades with vector directions, Φ, at least 20 deg away from the cardinal directions (N = 108).

**FIGURE 9:**
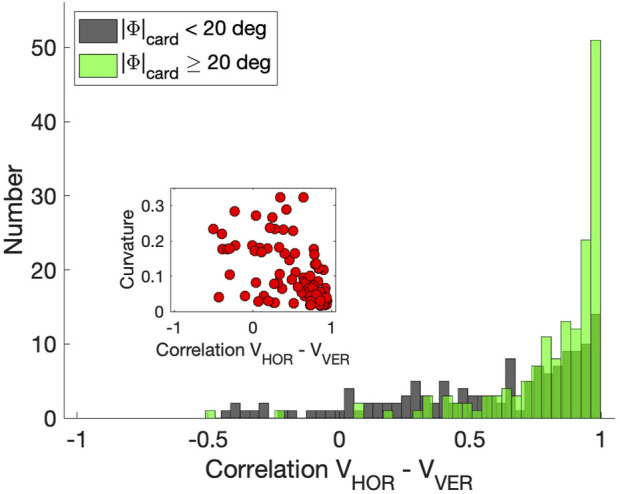
Correlations between the horizontal and vertical velocity profiles of the saccades from the continuous paradigm. Green histogram: oblique saccades, at least 20 deg away from the cardinal directions (N = 150). Gray historgram: saccades within 20 deg of the cardinal directions (N = 148). Inset: relation between the correlations and absolute curvature. Note that for straight saccades data points are found at [1,0].

Figure 9 shows these correlations as the green negatively skewed histogram with a clear peak near *r* = +1.0 (mean: *μ* = 0.82, std: *σ* = 0.23). The gray histogram shows the correlations for the saccades that remained closer to the cardinal axes; correlations now vary considerably more because one of the components is small, yielding a low signal-to-noise ratio (*μ* = 0.57, *σ* = 0.38). The inset suggests that the curvature measure and velocity-profile correlations correlate for the population of oblique saccades, (*r* = −0.56), although the straight saccades all cluster near (*r*, |*C*|) ≈ (1, 0). For the zero-initial paradigm this correlation was indeed insignificant as even a larger proportion of saccades was straight (no curvatures |*C*| > 0.15; see above, and Supplementary Material).

### 3.5 Component cross-coupling

**FIGURE 10:**
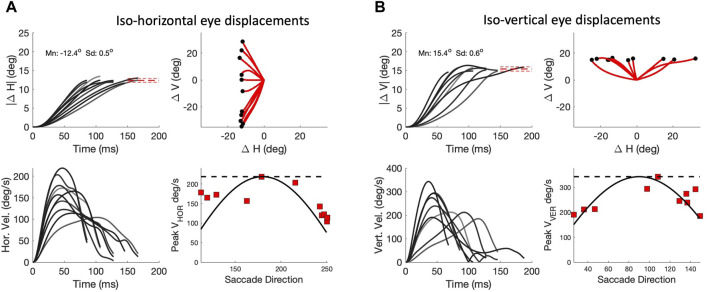
Component cross-coupling in oblique saccades (continuous paradigm). **(A)** Top-left: Ten oblique saccades with a fixed horizontal component of 12.4 deg. Top-right: 2D trajectories. Red-dashed lines: mean ± std of the end points. Lower-left: Horizontal velocity profiles. Note that the peak velocity and duration of this component vary strongly with the different oblique trajectories, indicating component cross-coupling. Lower right: peak velocity of the horizontal components varies systematically with the saccade direction. Solid line: cosine prediction of the common-source model (Eq. 12). Dashed line: prediction of the independent model (see text). **(B)** Ten oblique saccades with a fixed vertical component of 15.4 deg. Now the CS model predicts that the peak velocity of the vertical components varies as the sine of saccade direction (lower-right panel).

As a consequence of the high component correlations, the signals responsible for these components will have to be tightly coupled in order to synchronize all underlying motor commands. The duration of a horizontal component of a fixed amplitude, Δ*H*, should then match the duration of the vertical component, and consequently, its peak velocity should systematically depend on the saccade vector direction, Φ. The same holds for a fixed vertical component. Figure 10 analyses these properties for saccades of our biomimetic simulator. Figure 10A shows a selection of oblique saccades with a fixed leftward horizontal component of Δ*H* = -12.4 deg, with vectors varying widely in direction between 110 and 250 deg. It can be immediately appreciated that the horizontal components vary greatly in their durations between about 70 and 155 ms, while their associated velocity profiles vary substantially in shape and peak velocity from about 220 deg/s for the pure leftward saccade down to about 100 deg/s for the extreme downward oblique ones. The lower-right panel shows the relationship between the saccade direction and the peak velocity of the horizontal component, together with the predictions from two opposing models: the horizontal dashed line is from the *‘independent control model’*, which holds that the velocity components are controlled by independent, non-interacting saccade circuits. The solid line is the cosine curve of (12), predicted by the *‘common-source model’* of Van Gisbergen et al. (1985); Smit et al. (1990), described above. In Figure 10B a similar analysis is shown for oblique saccades with a fixed upward vertical component of Δ*V* = +15.4 deg. For these saccades, the common-source model predicts a sine-shaped relation for the component peak velocities. Clearly, the common-source model better accounts for the data than the independent control model for either component.

**FIGURE 11:**
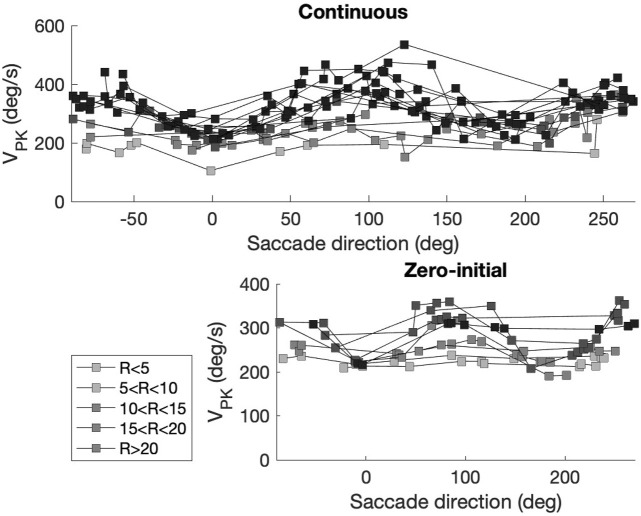
Peak velocity of the saccade vector varies with the saccade direction. Top: continuous paradigm. Bottom: zero-initial paradigm. Saccade amplitudes (R) were grouped into bins of at least nine responses (legend). Horizontal saccades (near 0 and 180 deg directions) are slower than vertical saccades (near 90 and 270 deg). Differences between values obtained for the two paradigms are due to additional changes in initial eye orientation for the continuous paradigm (e.g., Figures 5A; Figure 12). Note also that in the continuous paradigm saccade amplitudes covered a much wider range (up to 50 deg), thus reaching higher peak velocities than the zero-initial paradigm (amplitudes ≤30 deg).

Note that deviations from the common-source predictions can be observed in Figure 10 as well. These deviations are caused by three factors: First, the prediction assumes perfectly straight saccade trajectories, which is clearly not the case for all saccades (see Figure 8). Second, the predicted relationships are based on the simplifying assumption that the vectorial peak velocity is independent of saccade direction. Third, the prediction also assumes that the saccade peak velocity is independent of the eye’s initial orientation. However, also the latter two assumptions are violated, as the saccades generated by our biomimetic simulator resulted to be faster in vertical directions than in horizontal directions, which is illustrated in Figure 4 and further quantified in Figure 11 for the population of saccades, shown separately for five different amplitude bins and for both paradigms. A similar direction-dependency of human and monkey saccade main-sequence dynamics has been documented in detail by Van Gisbergen et al. (1985).

**FIGURE 12:**
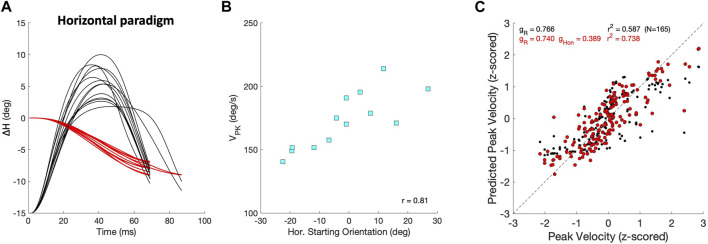
Saccade peak velocity also varies with initial eye orientation. **(A)** Thirteen equal-sized leftward saccades from the horizontal paradigm (red; ΔH = −7.9 ± 0.9 deg) that started from different initial eye orientations on the horizontal meridian (Methods). Black traces show the associated velocity profiles, all normalized with respect to the fastest saccade. Note that the peak velocities varied considerably. **(B)** The peak velocities of the saccades in **(A)** systematically depend on the initial horizontal eye orientation (r = 0.81), as leftward saccades were faster when starting from the right. **(C)** Regression results on the z-scores of peak velocity for all horizontal saccades with R ≥6 deg (Eq. 16). Black: V_PK_ only as function of amplitude yields r =0.77; red: as function of amplitude and initial eye orientation gives r =0.86. Parameters g_R_ and g_Hon_ are the partial correlation coefficients of the regressions.

The velocity profiles also varied with changes in the initial eye orientation, which partly accounts for differences in the dynamics and kinematics observed between the continuous and zero-initial paradigms. Figures 12A, B illustrates this property for a selected group of purely leftward horizontal saccades of identical size that were elicited by our Horizontal Continuous Paradigm from different initial eye orientations along the horizontal axis. Figure 12A shows the eye-orientation trajectories (red) and associated velocity profiles (black, normalized). The center panel in Figure 12B shows how their peak velocities changed as a function of the initial horizontal eye position. Typically, the peak velocity increased when the eye started from a contralateral position. In this example, the leftward saccades were faster when starting at rightward orientations. Conversely, rightward saccades were typically faster when starting from leftward eye orientations. In Figure 12C, we quantified the effect of initial horizontal eye orientation on the peak velocity by comparing two linear regressions (Eq. 16) on the dimensionless z-scores of amplitude, Δ*H*, and initial orientation, *H*_*ON*_, of 165 saccades whose amplitudes were ≥6 deg, for which peak velocity increased nearly linearly with amplitude:

$$\begin{array}{cc} {\hat{V}}_{PK} & =g_{R1}\Delta \hat{H}\\ {\hat{V}}_{PK} & =g_{R2}\Delta \hat{H}+g_{\mathrm{Hon}}{\hat{H}}_{ON}\\ & \text{where}\;\hat{z}\equiv \frac{z-\mu_{z}}{\sigma_{z}} \end{array}$$ (16)

with $g_{R1,2}$ and $g_{\mathrm{Hon}}$ the partial correlations of the regressions. Figure 12C provides the results of both regressions. While $g_{R1}\approx g_{R2}∼0.75$ , the contribution of initial eye orientation $\left(g_{\mathrm{Hon}}=0.39\right)$ was an independent factor that significantly increased the quality of the fit from *r* ^2^ = 0.59 to *r* ^2^ = 0.74 (*p* < 10^–6^).

Similar eye-position influences on the dynamics of saccadic gaze shifts have been reported in the psychophysical literature, e.g., in Robinson (2022); Van Opstal (2023).

### 3.6 Muscle synergies

Straight oblique trajectories (Figures 8–10) and a nonlinear main sequence (Figure 7) both result from the six synergistic command signals that drive the motors to generate the appropriate torques for the tendons. As the eye has only three rotational DOF, infinitely many possible control combinations of the 6 DOF system could generate identical saccade trajectories. For example, a horizontal rightward saccade could be elicited by having the four vertical/torsional muscles all inactivated, with the lateral and medial rectus muscles both activated such that the net result is a rightward rotation of the eye. Yet, the amount of co-contraction of the LR/MR and SO/IO/SR/IR muscles remains unspecified and could take on any combination as long as the total net torque corresponds to an appropriate rightward eye rotation. Our optimal control algorithm does not explicitly penalize the amount of co-contraction. It is therefore interesting to analyze how the different eye trajectories are actually implemented by this redundant control system.

**FIGURE 13:**
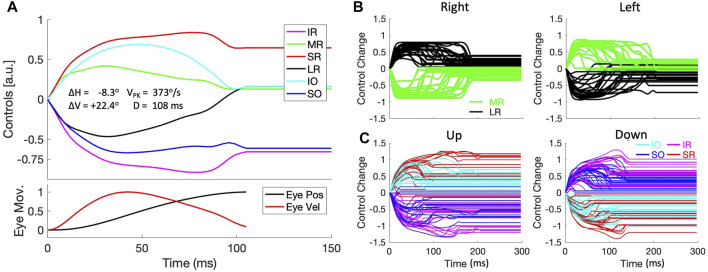
Muscle synergies (continuous paradigm). **(A)** Example of the six motor control signals for a saccade with a small leftward and a large upward displacement of the eye [R, Φ] = [23.9,110.3]^o^. Note that the six muscles are recruited as two antagonistic groups of three muscle pairs: the SR/IO/MR are activated, whereas the IR/SO/MR are inactivated re. pretension during the saccade. The patterns of the excited muscles resemble a pulse-step activity, while the inhibited group shows an anti-pulse-step activation. Note also the tight synchrony of the pulses with the saccade velocity profile (red trace in the bottom panel). **(B)** Control signals of the LR and MR antagonists for horizontal saccades, selected from the population of continuous saccade traces across the oculomotor range. The activities for the vertical/torsional muscles are not shown for clarity. **(C)** Control signals for the agonistic SR/IO (red/cyan) and IR/SO (magenta/blue) muscle pairs for vertical saccades. Here, the LR/MR activations have been omitted for clarity.

Figure 13A shows an example of the muscle activation patterns for a left-upward saccade with an amplitude of about 24 deg in a direction of 110 deg. The lower panel shows the vectorial change in eye orientation (black) and its instantaneous velocity (red) on normalized scale. The muscle control signals are all shown relative to their initial pretension, so that the activation of each muscle is shown as a change with respect to its tension at (0,0,0). Interestingly, the six muscles appear to be organized into two agonist and antagonist groups, formed by a positive change in activation of the MR/IO/SR muscles vs. a negative change for the LR/SO/IR, respectively. It can also be observed that the three agonist muscles show a pulse-step activation pattern, with the antagonists an anti-pulse/anti-step profile. The pulses and antipulses all end at the saccade offset, and seem to synchronize to a considerable degree their rapid increase or decrease at the start of the saccade, which convert to a more gradual change for all muscles near the moment of saccade peak velocity.

This pattern resulted to be representative for all saccades in both eye-movement paradigms. Figure 13B shows the antagonistic behavior of the lateral (black) and medial (green) recti for all near-horizontal rightward and leftward saccades selected from the continuous data base (directions within 10 deg from horizontal). It can be appreciated that the pulse-durations of the muscle pair are synchronized, but also that these pulse-durations vary considerably from saccade to saccade. The latter point underlines the fact that despite the considerable nonlinearities that determine the mechanics of the eye plant, the main-sequence nonlinearity of Figure 7 is already observable in the pulse control signals of the muscles. Figure 13C shows the activations of the vertical/torsional muscles for near-vertical (up/down) saccades (directions within 10 deg from the vertical axis). Here it can be seen that the SR/IO and SO/IR muscles form agonistic pairs for upward and downward saccades, respectively. Similarly, SR/IR and SO/IO act antagonistically for these eye movements. Note also that the maximum changes in muscle activation for the vertical/torsional muscles reach higher levels than the horizontal muscles, which underlies the result that the vertical saccades of our biomimetic eye reached higher peak velocities than horizontal saccades (Figure 11). Similar results were obtained for the zero-initial paradigm (Supplementary Material).

**FIGURE 14:**
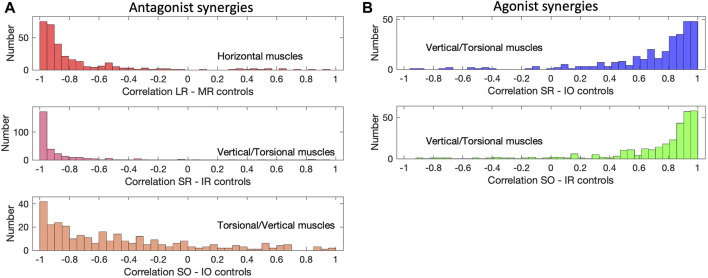
Muscle correlations (continuous paradigm). **(A)** The activation signals of antagonistic muscle pairs are expected to be negatively correlated. This is indeed observed for the LR/MR pair, the SR/IR pair and the SO/IO pair (see also Figures 13A–C). The latter has a wider distribution with some lower correlations, which is caused by the small range of torsional movements due to Listing’s Law (Figure 6B). **(B)** For agonists one expects positive correlations, shown here for the SR/IO muscle pair and the SO/IO muscles, which work as agonists for vertical saccades (see, e.g., Figure 13C).

To quantify the amount of synchronization among the muscle activation patterns, we calculated the correlations between the muscle control signals during each saccade. It is then expected that, for agonist muscles, these correlations should be positive and, ideally, close to one, whereas for antagonists they should be negative, ideally close to minus one. Figure 14 shows the distributions of these correlations between five different muscle pairs for all 298 saccades of the continuous paradigm, inspired by the patterns shown in Figure 13. Figure 14A shows the three groups of muscles that were identified as antagonists: the LR/MR (top), the SR/IR (center), and the SO/IO (bottom). Note that the former two pairs indeed have most of their correlations close to −1.0, while the SO/IO pair seems to be more variable. The latter is due the fact that the saccades obey Listing’s Law (Figure 6B) and therefore the saccade trajectories show only a limited amount of cyclotorsion. Some variability is also seen in the LR/MR pair, which is due to the low signal-to-noise ratio for these muscles for near-vertical saccades. Figure 14B quantifies the correlations for two agonist muscle pairings: SR/IO and SO/IR. These correlations indeed peak close to +1.0, where here the lower correlations result from their weak involvement for near-horizontal saccades.

Taken together, the control algorithm for our biomimetic eye yielded oculomotor behaviors and muscle-control signals that highly resembled those observed for the primate saccadic eye-movement system (Fuchs and Luschei, 1970; Fuchs and Luschei, 1971; Robinson and Keller (1972); Hepp and Henn (1985); Suzuki et al. (1999)).

### 3.7 Motor code for 3D eye movements

The pulse in the pulse-step motor commands is related to the dynamics of the eye movement, but it could encode the eye’s *angular velocity* around the axis of rotation, ***ω*** (*t*), the 2D dynamic change in orientation (also called the *coordinate velocity*), $\dot{r}(t)$ (e.g., Van Opstal et al. (1991); Van Opstal et al. (1996); Klier et al. (2006); Suzuki et al. (1999)), or some hybrid combination of these signals. Note that the coordinate velocity vector is confined to LP (since ***r*** is in LP), but that the angular velocity vector tilts out of the plane whenever the coordinate velocity-, $\dot{r}(t)$ , and initial eye orientation vectors, ***r***_*on*_, are not parallel, because of the vector cross product in (3). Here we show how our simulator can be used to investigate this question in similar ways as has been done in neurophysiological experiments.

Suppose that the eye has a vertical initial orientation, *ϕ*_0_ (i.e., $r_{on}=\tan\left(\phi_{0}/2\right)\cdot \hat{y}$ ), and that a horizontal eye movement is made in LP, described by $\dot{r}(t)={\dot{\theta }}_{H}(t)\cdot \hat{z}$ , with ${\dot{\theta }}_{H}(t)$ its horizontal velocity profile.

The eye’s angular velocity is then given in Eq. 17 as:

$$\omega \left(t\right)\approx 2\left(\dot{r}\left(t\right)+r_{on}\times \dot{r}\left(t\right)\right)=2{\dot{\theta }}_{H}\left(t\right)\left(\hat{z}+\tan\left(\phi_{0}/2\right)\hat{x}\right)$$ (17)

which specifies a fixed-axis rotation of the eye, and the angle, *ρ*, between ***ω*** (*t*) and $\dot{r}\left(t\right)$ is

$$\rho =\phi_{0}/2$$ (18)

Eq. 18 is known as the ‘half-angle rule’ (Tweed and Vilis (1987); Van Opstal et al. (1991); Klier et al. (2006); see Supplementary Figure S10B).

If the motor signals encode the angular velocity vector of the eye, one expects that the pulse control signal in the vertical-torsional system for *horizontal* saccades would depend in a systematic (linear) way on the vertical initial eye orientation. In the Supplementary Figure S9, 10) we illustrate this principle for our biomimetic system. These results indeed indicate that our 6 DOF system programs a velocity vector that accounts for the non-abelian properties of 3D rigid-body rotations.

One can also ask what happens if the horizontal eye movement is not programmed by the brain, but instead elicited by electrical stimulation of the abducens nerve, which directly innervates the LR muscle. Exactly this experiment was conducted by Klier et al. (2006). They reasoned that if LL is implemented by a neural control, and not by the biomechanics of the plant, the stimulation-induced movement should violate LL, as it would elicit a signal very close to $\dot{r}$ , instead of the required pulse from all agonistic muscles that will yield ***ω*** (Hepp et al., 1989). Instead, the authors showed that the stimulation-induced eye movement obeyed the half-angle rule, i.e., the eye stayed in LP also during the stimulation, from which they concluded that LL has a biomechanical origin (at least for horizontal eye movements).

To replicate this experiment with the biomimetic eye, we stimulated either the LR muscle, or the MR muscle, of the physical simulator from different initial vertical eye orientations, all in LP (reached by 13 selected trajectories from the zero-initial paradigm). We tested whether the resulting coordinate velocities would stay in LP during LR/MR stimulation, like in Klier et al. (2006). The coordinate velocity (Eq. 19) is calculated from the eye’s instantaneous angular velocity and orientation (Eqs 3, 17), which are both obtained from the state of the simulator, by (Hepp, 1990):

$$2\dot{r}=\omega +\omega \times r+\left(\omega •r\right)r$$ (19)

**FIGURE 15:**
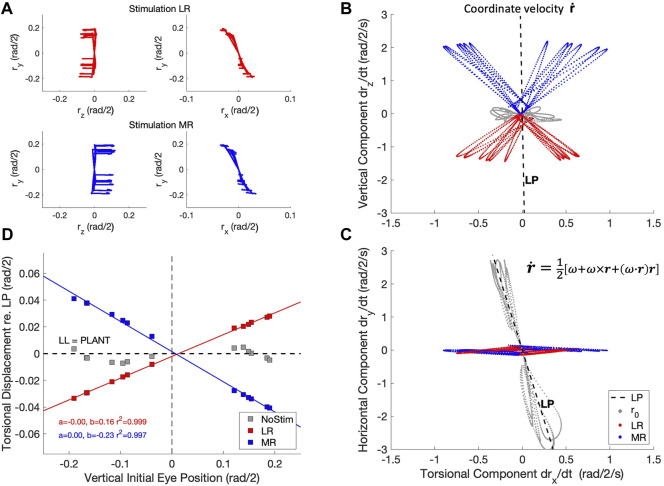
The half-angle rule is not implemented by the oculomotor plant of the robotic eye, contrary to the results from Klier et al. (2006) in monkey. **(A)** Experimental paradigm: an eye movement is made from straight ahead to a vertical target location (see also Figure 4), after which either the LR muscle (red traces) or the MR muscle (blue traces) is stimulated by a Gaussian pulse with a width of 21 ms. This elicits a rightward (top-left) or leftward (bottom-left) eye movement from 13 different elevations with a small clockwise or counterclockwise torsional displacement (Hepp et al., 1989). The right-hand panels show an expanded (r_x_, r_y_) view of the traces in the laboratory frame (cf. Figure 6B). **(B, C)** The horizontal movements elicited by the stimulation produced a coordinate velocity, $\dot{r}$ , that clearly deviates from Listing’s plane. Red: LR stimulation; blue: MR stimulation. Light-grey traces are the vertical eye movements before the stimulation, and they define Listing’s Plane (dashed black lines, LP). **(D)** The total deviation from LP, $\Delta r_{x}=\int {\dot{r}}_{x}dt$ , depends linearly on the initial vertical eye orientation. If the plant would have implemented Listing’s Law, the data should scatter around the black-dashed line (and the angular velocity vector, **ω**, of the movements (not shown) would have followed the half-angle rule; see also Supplementary Figure S10).

Figure 15 shows the result of this simulated experiment. In contrast to Klier et al. (2006), LR or MR stimulation of the biomimetic eye clearly violates LL, as it brings the eye out of Listing’s Plane in a way that varies remarkably linearly with the initial vertical eye orientation. Linear regression yielded:

$$\begin{array}{ccc} \Delta r_{x}^{LR}=0.16\cdot r_{y}^{on} & & \\ \;\Delta r_{x}^{MR}=-0.23\cdot r_{y}^{on} & & \end{array}$$

with *r* ^2^ > 0.995.

In the (Supplementary Figure S8) we show the results of a similar stimulation experiment applied to the trochlear nucleus fibers that directly innervate the SO muscle, from different initial horizontal eye positions. The stimulation of this experiment again shows a tight linear relation between the initial position and the total amount of cyclo-torsion. To our knowledge, such an experiment has not yet been performed.

## 4 Discussion

### 4.1 Main results

We analyzed the eye-movement properties of a 6 DOF biomimetic robotic eye in detail, following similar analysis approaches as have been applied to real human and monkey eye movement data.

Our analysis demonstrates that, despite several simplifications discussed below, many properties closely resemble those of human and monkey saccades. The eye-movement dynamics show single-peaked velocity profiles that obey straight-line relationships between the saccade amplitude and duration (Figure 7A), and the product of peak eye velocity and duration (Figure 7B). As a result, the peak velocity saturates as a function of saccade amplitude (Figure 7C). Together, these dynamic properties functionally define saccades (Bahill et al. (1975); Van Opstal and Van Gisbergen (1987); Robinson (2022)). The latter relation has considerable variability, which could be largely attributed to the direction-dependence of the *R* − *D* and *R* − *V*_*PK*_ relations (Figure 11), and their dependence on the initial eye orientation (Figure 12).

A detailed assessment of the curvature of the eye-movement trajectories shows that the majority of oblique saccades was virtually straight, with only a minority of about 7% substantially curved (Figure 8B). The sign and amount of curvature varied systematically with the saccade direction and also had a strong contribution from variation in the initial eye orientation (Figure 8C); these aspects are similar to those reported for human saccades (Smit and Van Gisbergen, 1990).

As a consequence of the straight trajectories, the horizontal and vertical velocity profiles are scaled versions of each other, leading to a considerable amount of cross-coupling between the components, like stretching of the duration of the smaller component to match the longer component. The analysis demonstrates that these data cannot be accounted for by an independent control strategy of the motor drives. In such a control, the velocity profiles of the individual components should not depend on saccade direction. Instead, the data more closely followed the predictions of the common-source model, which assumes a central vectorial velocity generator that is subsequently decomposed into its vector components (Figures 9, 10; Van Gisbergen et al. (1985); Smit et al. (1990)). Since the six motors are physically independent, this vectorial control signal is an acquired strategy that emerged from the optimal control. Neurophysiological studies have provided support for the idea that the midbrain Superior Colliculus could be responsible for sending this central vectorial velocity command to the brainstem pulse generators (Van Opstal, 2023).

The 3D orientation of the biomimetic eye obeyed Listing’s Law with a variability around the best-fit plane of less than a degree, which is in line with results from voluntary eye movements reported for human and non-human primates. The orientation of the right-eye’s plane was tilted slightly leftward by about 7 deg in the (*x*, *y*) plane, so that its primary position was about 7 deg to the right of the straight-ahead laboratory frame. This particular location of the primary position could be related to the fact that the insertion points of four of the muscles were at the annulus of Zinn (Figure 1B), and therefore their pulling directions, were shifted leftward with respect to the center of the right eye. It also explains why the static tension in the LR muscle at fixation is slightly higher than that of the MR muscle (Figure 14B), as its length at the (0,0,0) position is slightly longer.

Interestingly, the control of the six muscles became organized in clear agonist-antagonist pairs (Figure 14; Sherrington (1906)). For oblique saccades, the three agonists were LR-SR-IO and MR-IR-SO. The antagonists were LR-MR for horizontal, and SR-IR with IO-SO and SR-IO with IR-SO for the vertical/torsional components (Robinson, 1975; Miller and Robinson, 1984).

The signals of the agonists could be succinctly described as pulse-step controls, where the pulse serves to overcome the overdamped nature of the plant (Robinson (1964); Robinson (2022)). The antagonists followed the inverse behavior: a rapid decline in tension during the eye movement, followed by a step increase to the new equilibrium level, which was typically lower than at the start of the saccade (Figure 13). These motor control patterns have been observed in all oculomotor neuron pools of abducens (nVI), oculomotor (nIII) and trochlear (nIV) nuclei in the monkey brainstem (Fuchs and Luschei, 1970; Fuchs and Luschei, 1971; Robinson and Keller (1972); Hepp and Henn (1985); Suzuki et al. (1999)). As also these antagonistic and pulse-step behaviors were not explicitly pre-programmed in the cost functional, they must have all emerged from the optimal control.

### 4.2 Plant mechanics and simplifications

Despite the many similarities of the eye movements produced by the simulated biomimetic eye with human saccades, there are also clear differences with results from the literature. A prominent difference is illustrated in Figure 15. Our simulation of the LR- (here we also added MR-) stimulation experiment of Klier et al. (2006) shows that our 6DOF system does not implement Listing’s Law through plant mechanics. Instead, upon LR/MR stimulation, the eye clearly violates LL, as the resulting coordinate velocity of the eye attains 3DOF (Figures 15B,C). Interestingly, the relationship between the amount of accumulated torsion and the initial vertical gaze angle was strikingly linear (Figure 15D). This strongly contrasts with the results from Klier et al. (2006) who demonstrated that LL is still obeyed during LR stimulation, presumably by a precise positioning of soft-tissue ‘pulleys’ (Quaia and Optican, 1998; Demer, 2006) for the LR and MR antagonists, which forces the eye’s angular velocity axis to tilt out of LP by half the angle of the vertical gaze direction (the ‘half-angle rule’). This prediction assumes that the stimulation-induced motor command from the LR muscle is a purely horizontal velocity signal, independent of the vertical initial position: ${\dot{r}}_{LR}∼v_{z}\cdot \hat{z}$ . Under natural conditions, a horizontal saccade will have to be accompanied by a vertical-position dependent torsional control signal from the vertical/torsional muscles, which is indeed the case for the pulse-step commands of the Continuous Paradigm (Supplementary Figure S10B). The discrepancy of our result with the Klier et al. (2006) findings may be resolved by adding a set of pulleys for the LR/MR pair (Schnabolk and Raphan, 1994; Quaia and Optican, 1998; Misslich and Tweed, 2001; Demer, 2006; Lee et al., 2007), but we here deliberately refrained from doing so, as our main aim was to test the model’s possibilities and properties without introducing additional mechanical assumptions. Indeed, if the eye is brought out of LP through torsional-vestibular stimulation, electrical LR stimulation still resulted to induce the half-angle rule, even though it is now an inappropriate response, not in line with normal behavior Klier et al. (2012). Thus, there is a clear 3D contribution to the neural control of eye movements, where LL may serve as the default strategy through a simple biomechanical implementation (Tweed et al., 1999; Klier et al., 2012).

A second difference with human behavioural data is that the velocity profiles of the model, illustrated in Figure 4, are less skewed and peaked than reported in the literature (Van Opstal and Van Gisbergen, 1987). Human saccade-velocity profiles are negatively skewed, for which the moment of peak velocity is roughly fixed for all saccade amplitudes (at ∼20–25 ms). In contrast, the profiles generated by the model are more symmetrical. In Cardoso (2019) we showed that by including multiplicative motor noise in the controls (i.e., $\tilde{u}∼{\left(1+\varepsilon \right)}^{T}\cdot u$ , with ***ɛ*** (0, *σ*) Gaussian noise), the velocity profiles become more skewed with sharper peaks. Moreover, the energy cost, *J*_*E*_(*D*), in the cost functional (Supplementary Material) can then be eliminated, as the quadratic control term then emerges from the accuracy cost, *J*_*A*_(*D*) (Shadmehr and Mussa-Ivaldi, 2012).

Other simplifications in our simulator are: (i) all muscles had the same elasticity and were treated as simple linear springs, which is not the case for real muscle (Quaia et al., 2011); (ii) the muscle trajectories were determined by the insertion points only, defining straight lines that sometimes could intersect the globe, instead of wrapping around it; (iii) the muscles were modeled by single-fiber tendons, rather than multiple-fiber elements that are partially fixed to a surface on the globe to prevent muscle side-slip (Robinson, 1975; Miller and Robinson, 1984), and (iv) apart from the SO and IO, we did not include additional pulleys to change muscle trajectories.

Whether pulleys ensure Listing’s Law for all eye muscles and for eye movements in all directions, however, remains unclear. Unfortunately, direct stimulation of muscle fibers is not possible for the SR, MR, IR and IO, as they all originate in the oculomotor nucleus (nIII) without a clear neuro-anatomical topography. The only other muscle for which this type of experiment could be performed would be the SO (innervated by the trochlear nerve, nIV), e.g., for different horizontal initial eye orientations, but to our knowledge, such an experiment has not been performed. With our simulator, however, it is straightforward to simulate the result, which is shown in Supplementary Figure S8). Clearly, as this muscle’s pulling force is not in LP, but in the vertical/torsional direction, the presence or absence of LL-related pulleys (besides the trochlea in the eye socket, Figure 1A) that ensure the half-angle tilt of the angular velocity axis could still be tested once the effect of SO stimulation from primary position (i.e., 7 deg to the right) is known.

### 4.3 Pretension

A nontrivial problem in the saccadic control of six muscles is the danger of *‘slack’*, which would occur if the controlled tension of an antagonist would go negative. In such a case, the muscle is (albeit briefly) out of control, which is clearly undesirable. We avoided this problem by providing a fixed level of pretension to all muscles when the eye looks at (0,0,0) (Javanmard Alitappeh et al. (2023); Supplementary Material). We obtained a suitable pretension level by trial and error, but it is conceivable that including a force constraint in the cost functional, an optimal pretension that minimizes the amount of co-contraction, which at the same time avoids slack, may be found by the optimal control. In a previous study, with a 3DOF prototype of the biomimetic eye, we found that a quadratic force constraint across all fixation positions also eliminated the need to specify the final goal’s cyclo-torsion at zero to keep the eye in Listing’s Plane (John et al. (2021). How to extend this requirement to the 6DOF system is left for future study.

### 4.4 Conclusion and future work

The 6DOF biomimetic model of the eye can be used to gain a better understanding of the neurophysiological and biomechanical factors that explain eye-movement behaviors over the full 3D oculomotor range. Conversely, use of an accurate simulator is also useful to finetune and test potential robotic applications of the system, or to quickly analyse the effects of changes in the design. We currently work on an improved implementation that will significantly reduce the training and computational time of the system from 180 s down to ±30 ms. The modelling is not exclusively confined to rapid saccades, but can in principle be extended to smooth pursuit eye movements, and/or to ocular nystagmus evoked by vestibular stimulation as well. For pursuit, it would suffice to also specify the target motion in the 6D goal vector, as the overall cost will still be dominated by speed-accuracy trade-off. For involuntary eye movements like vestibular-evoked nystagmus, for binocular control like in vergence, or for combined eye-head gaze shifts, the motor behaviors are no longer dictated by Listing’s Law but by 2D task-dependent constraints that are governed by Donders’ Law and by visual requirements (Tweed et al. (1998); Tweed et al. (1999); Misslich and Tweed (2001); Klier et al. (2012)). Thus, for these more complex sensory-motor synergies, different task-dependent costs or cost weightings will have to be considered, like perceived visual orientation, or motor effort. Moreover, including the head for gaze control further extends the number of degrees of freedom for the system: not only does the head allow for combined rotations and translations, but also the larger number of muscles and their insertions allow for a richer repertoire of behaviors. It will be interesting to test whether the optimal control algorithm with a minimum number of costs may lead to realistic muscle controls and behaviors, such as demonstrated here for the 6 DOF biomimetic eye. Optimization of gaze control may further benefit from the inclusion of a retina-inspired foveate camera, which will automatically enforce the system to rapidly explore the visual environment through accurate saccades.

## Data Availability

The datasets presented in this study can be found in online repositories. The names of the repository/repositories and accession number(s) can be found below: Donders institute repository https://data.ru.nl/collections/di/dcn/DSC_626870_0003_600.

## Footnotes

1 The fact that *J*_*D*_ is non-convex does not ensure a convex *J*_*MOV*_ for all possible choices of *λ*_*α*_.
