# Supplementary material for: Realistic 3D human saccades generated by a 6-DOF biomimetic robotic eye under optimal control
Source: Front Robot AI. 2024 May 21;11:1393637. doi: 10.3389/frobt.2024.1393637 (PMC11149426; doi:10.3389/frobt.2024.1393637)
Supplement: Supplementary file 1 [file DataSheet1.PDF]

Supplementary Material to: Realistic 3D Human Saccades Generated by a 6-DOF Biomimetic Robotic Eye Under Optimal Control

1 SUPPLEMENTARY DATA

This Supplementary Material provides a brief account of the physics background of the robotic prototype and its numerical implementation. More specific details can be found in Javanmard Alitappeh et al. (2023). We here also present the results of the *Zero-Initial Paradigm* described in the paper, and the results of a simulation for a direct superior-oblique electrical stimulation experiment. The formats of the figures are similar as the associated ones in the manuscript for the continuous saccade paradigm.

2 PHYSICS MODEL, COSTS FOR OPTIMAL CONTROL, AND NARX MODEL

2.1 The Model and its Simulator

We define $\boldsymbol{u}_{0:D}$ as a 6D sequence of input motor commands for a saccade trajectory, where $\boldsymbol{u} \equiv [u_{IR}, u_{MR}, u_{SR}, u_{LR}, u_{IO}, u_{SO}]^T$, and $\boldsymbol{x}$ is the system's 6D state vector representing the 3D orientation, $\boldsymbol{r}$, and angular velocity, $\boldsymbol{\omega}$, of the eye: $\boldsymbol{x} \equiv [\boldsymbol{r}, \boldsymbol{\omega}] = [r_x, r_y, r_z, \omega_x, \omega_y, \omega_z]^T$.

In the physical model, the spherical eye was subjected to Newton-Euler's rigid body equation of angular motion, resulting from the torques generated by the six tendons:

$$\mathbf{I}\boldsymbol{\alpha} = \boldsymbol{\tau}_{net}(\boldsymbol{x}, \boldsymbol{u}) - \boldsymbol{\omega} \times \mathbf{I}\boldsymbol{\omega} \tag{S1}$$

Here, $\boldsymbol{\alpha}$ is the eye's angular acceleration, diag$(\mathbf{I}) = [4.8, 4.3, 4.8] \times 10^{-4}$ kg·m^2 is its tensor of inertia, $\boldsymbol{\omega}$ the eye's angular velocity; $\times$ is the vector cross product, and $\boldsymbol{\tau}_{net}$ the total torque on the eye. The latter is generated by dynamic friction, $\boldsymbol{\tau}_{fr}$, and and by elasticity, $\boldsymbol{\tau}_{el}$:

$$\boldsymbol{\tau}_{net} = \boldsymbol{\tau}_{el} + \boldsymbol{\tau}_{fr} = \sum_{m=1}^{6} \boldsymbol{\tau}_m - \mathbf{D}\boldsymbol{\omega} \tag{S2}$$

with diag$(\mathbf{D})$ = [0.04,0.04,0.04] Nms the eye's damping matrix, subscript $m \in$ [IR,MR,SR,LR,IO,SO], and each muscle's torque is

$$\boldsymbol{\tau}_m = \mathbf{Q}_m \times \mathbf{f}_m \tag{S3}$$

Here, $\mathbf{Q}_m$ is the muscle's insertion point on the eye, and $\mathbf{f}_m$ is the force applied by the muscle on the eye that depends on the state $\boldsymbol{x}$ and on the control input $\boldsymbol{u}$.

Tendons were modeled as linear elastic strings, all with elasticity $k = 20$ N. The (dynamic) elastic force, $\mathbf{f}_m$, then depends on the instantaneous cable length, l_m, which varies with the rotation of the motors ($\boldsymbol{u}$) and the current orientation of the eye (in state $\boldsymbol{x}$), by:

$$l_m(\boldsymbol{r}, \boldsymbol{u}) = \|\mathbf{Q}_m(\boldsymbol{r}) - \mathbf{P}_m\| + r_{sp} \cdot u_m \tag{S4}$$

with $\mathbf{Q}_m$ and $\mathbf{P}_m$ the (eye- and head-)insertion points of tendon m (Table S1), r_{sp} the radius of the spindle, and u_m the rotation angle (in radians) of the spindle for cable m. The resulting dynamic elastic force is determined by Hooke's law:

$$\mathbf{f}_m = \frac{k}{l_{0m}}(l_m(\boldsymbol{r}, \boldsymbol{u}) + r_{sp} \cdot u_{m,0} - l_{0m})\hat{\boldsymbol{\phi}}_{\boldsymbol{m}} \tag{S5}$$

with l_{0m} the tendon's rest length (Table S1), and $\hat{\boldsymbol{\phi}}_{\boldsymbol{m}}$ the unit vector in the direction of the force. The term $r_{sp} \cdot u_{m,0}$ sets the pretension, which prevents the muscle from going slack. We found that $u_{m,0} \sim 2$ rad yielded satisfactory results.

2.1.1 Cable insertions

Table S1 provides the coordinates of the eye-centered and head-centered insertion points of the six tendons. These insertion points were appropriately scaled from the anatomical data provided by Miller and Robinson (1984); John et al. (2023). The eye's diameter was 80 mm.

**Muscles**	IR	SR	MR	LR	IO	SO
Insertion(eye)						
Q_x	-2	-2	7.7	7.7	-11.2	-11.8
Q_y	-1.4	0.8	39.3	-39.3	-1.4	-1.2
Q_z	40	-40	0	0	38.3	-38.1
Insertion(head)						
P_x	-100.1	-100.1	-100.1	-100.1	45	45
P_y	40.7	40.7	51.6	29.6	37.5	37.5
P_z	7.8	-14.9	-3.5	-3.5	62	-62
Rest Length	111.5	108.8	108.6	127.9	72.3	72.7

Table S1. *Locations of the cable insertion points at the eye surface and at the head with the eye in the equilibrium position. All measures are in mm. In the right-handed Cartesian reference frame, the center of the eye is at (0, 0, 0) mm. Four muscles (IR, SR, LR, MR) have their head-insertions at the annulus of Zinn (with $x \sim -100$ mm, and $y \sim +30 - 50$ mm); the SO and IO are guided through pulleys. Muscle lengths are l_{0m}. Note that the LR muscle is longer than the MR muscle.*

2.2 Optimal control

The optimization function $J(\boldsymbol{x}, \boldsymbol{u}, D)$ for a selected goal $\boldsymbol{x}_G \equiv [0, G_y, G_z, 0, 0, 0]$, and movement duration, D, can be written as follows:

$$\begin{gathered}\mathrm{U}^* = \underset{\boldsymbol{u}}{\text{argmin}} \;\; J(\boldsymbol{x}, \boldsymbol{u}, D) = \sum_{\alpha \in \{D,A,E\}} \lambda_\alpha J_\alpha(\boldsymbol{x}, \boldsymbol{u}, D) \\ \textbf{subject to:} \quad \boldsymbol{x}_{t+1} = f(\boldsymbol{x}_t, \boldsymbol{u}_t), \quad t = 0, 1, ..., D\end{gathered} \tag{S6}$$

where $f(\boldsymbol{x}_t, \boldsymbol{u}_t)$ describes the dynamics of the eye-plant (the simulation function; (S1-S5). In this formulation, J_α, indicates each of three costs: *Duration* (J_D), *Accuracy* (J_A), and *Energy* (J_E), defined hereafter. Further, $\boldsymbol{x}_{t+1}$ is the next state of the eye, which is estimated by the NARX approximator (see below).

1. Duration cost ($\boldsymbol{J_D}$)

The shorter the duration of the saccade, the better. The duration cost is defined by the hyperbolic discount-of-reward function (Shadmehr et al. (2010)):

$$J_D(D) = 1 - \frac{1}{1 + \beta D} \tag{S7}$$

with β a fixed parameter. The shorter duration D, the lower $J_D(D)$.

2. Accuracy cost ($\boldsymbol{J_A}$)

An ideal eye movement should reach to the goal state $\boldsymbol{x}_G$ at time D as accurately as possible. Therefore, in this cost (J_A) we compute the Euclidean norm of the difference between the eye final state and the desired one.

$$J_A(\boldsymbol{x}) = \sum_{t=1}^{W} ||\boldsymbol{x}_G - \boldsymbol{x}_{D+t}||^2. \tag{S8}$$

where, W is a time window to penalize any change before the goal state ($W = 5$).

3. Energy cost ($\boldsymbol{J_E}$)

During a saccadic eye movement, one other cost is the energy consumption by the muscles, so that J_E indicates the total energy of the motors, which follows Shadmehr and Mussa-Ivaldi (2012):

$$J_E(\boldsymbol{u}) = \sum_{t=1}^{D} ||\boldsymbol{u}_t - \boldsymbol{u}_{t-1}||^2 \tag{S9}$$

Because an analytical solution for the minimization problem (S6) does not exist, we obtained suitable weighting coefficients $\lambda_{D,A,E} = [1, 25, 0.05]$ by trial and error, by demanding that

$$J_{TOT}(D) = \sum_{\alpha} \lambda_\alpha J_\alpha(\boldsymbol{x}, \boldsymbol{u}_{opt}(D), D) \quad \text{is a convex function of } D \tag{S10}$$

with $\boldsymbol{u}_{opt}(D)$ the optimal command found with the NARX network for a given saccade duration $D \in [30 : 20 : 210]$ ms.

2.3 The NARX approximator

To learn the nonlinear dynamics of the biomimetic eye in a model-free and computationally efficient way, we approximated the system by training a nonlinear autoregressive network with exogenous inputs (NARX) to a long sequence of pseudo-random input-output data (Fig. 3A in the paper). Here we applied this procedure to the nonlinear simulator of the eye described above, but note that it can also be applied to the input-output relations measured from the real physical robotic prototype.

The network's operation can be written in discretized time as:

$$\boldsymbol{x}_{t+1} = \tilde{f}(\boldsymbol{x}_{t-n_x:t}, \boldsymbol{u}_{t-n_u:t}) \tag{S11}$$

where the next (predicted) state of the eye, $\boldsymbol{x}_{t+1}$, depends in a nonlinear way, $\tilde{f}(\cdot)$, on the inputs from a sequence of $n_x = 3$ previous samples of the 6DOF state, $\boldsymbol{x}_{t-n_x:t}$, and of $n_u = 1$ samples of the 6DOF

motor commands $\boldsymbol{u}_{t-n_u:t}$ (these had a three times lower sampling rate than the state). After training, $\tilde{f}(\boldsymbol{x}_{t-n_x:t}, \boldsymbol{u}_{t-n_u:t})$ approximates the nonlinear plant dynamics, $f(\boldsymbol{x}_t, \boldsymbol{u}_t)$.

In the network, the input arrays were fed to a hidden layer of $n_h = 55$ units (with sigmoid input-output characteristics), and their outputs were in turn forwarded to the output layer, consisting of 6 linear units representing the 6D vector of the predicted output state of the eye. The 1711 weights (6x3x55 + 6x55 + 55x6 and 61 biases) that connected the layers were trained by the Levenberg-Marquardt backpropagation algorithm.

We used the trained NARX network to find the optimal control for the eye. Once the optimal control was obtained, it was fed to the actual nonlinear physical simulator for further study of the eye-movements as described in the paper. The full procedure is schematically illustrated in Figure 3 of the manuscript.

3 SUPPLEMENTARY FIGURES

3.1 Accuracy and 3D kinematics

Figure S1 illustrates the endpoint accuracies of the 199 saccade vectors.

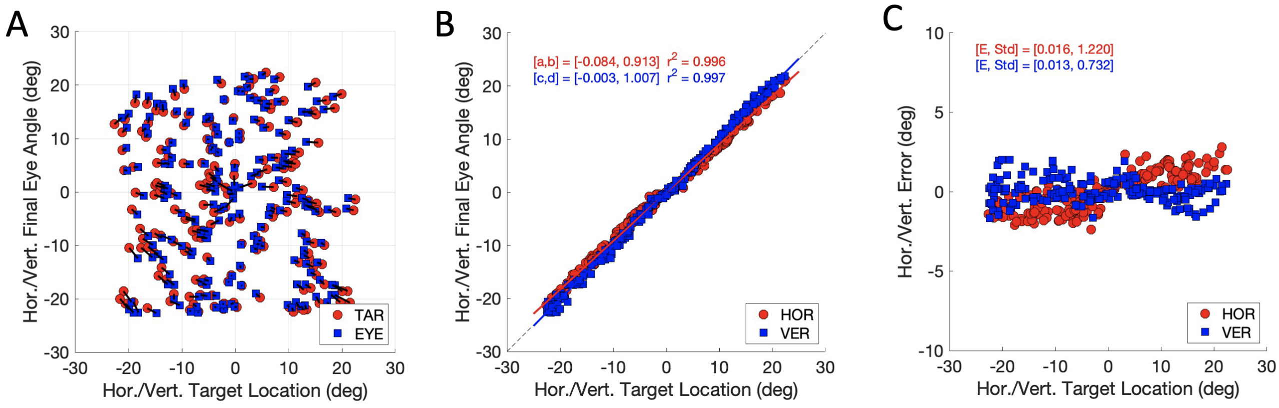

Figure S1. *Accuracy of the zero-initial saccades:* ***(A)*** *target locations vs. saccade endpoints,* ***(B)*** *regression of the stimulus-response relations, and* ***(C)*** *signed errors for the horizontal and vertical components.*

In Figure S2 it is shown that the saccade trajectories are well described by Listing's law and that the parameters of the best-fit plane correspond well to those of the continuous paradigm. The plane is tilted in the xy projection of the laboratory frame. The Listing coordinates (and hence the primary position) are found by a rightward rotation of $\arctan(-0.117) = 6.7$ deg around the z-axis. The variability of the trajectories in the continuous paradigm is larger than for the zero-initial paradigm: the width of LP in the zero-initial paradigm is only 0.3 deg vs. 0.8 deg for the continuous paradigm.

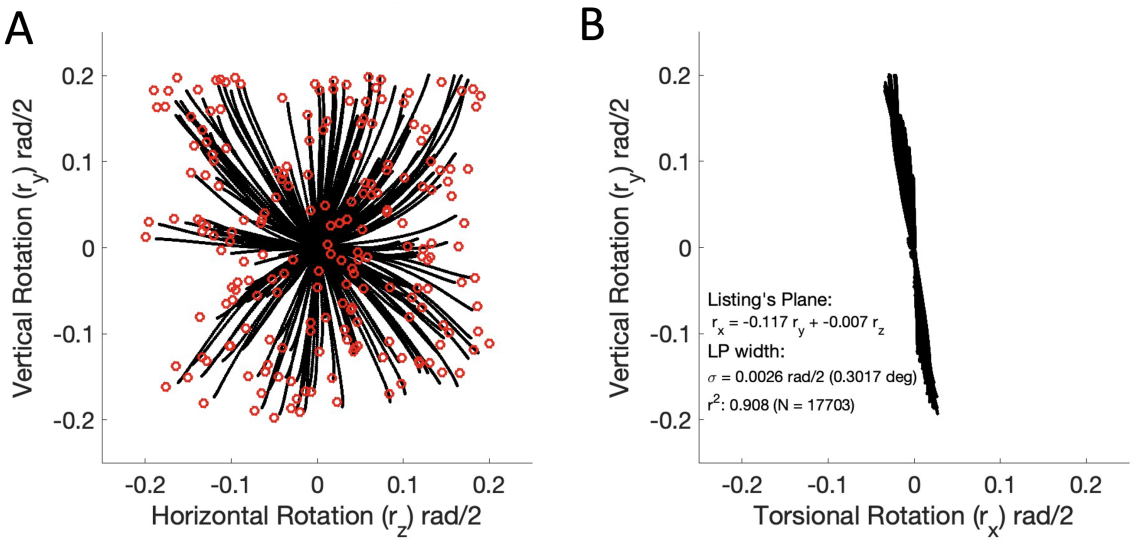

Figure S2. *Listing's Law describes the 3D rotational kinematics well.*

3.2 Main-sequence dynamics

The zero-initial saccades obey the nonlinear main-sequence dynamics, as can be seen in Fig. S3. Especially the shape relation of $V_{PK} \cdot D = k \cdot R$ is very tight, with $r^2 = 0.945$. Note, that in the zero-initial paradigm there is no variability caused by changes in initial eye orientation, and also that the amplitude range is smaller (about 30 deg) when compared to the continuous paradigm (about 50 deg). The asymptotic peak velocity for the saccade population is estimated at $V_{PK}(\infty) = 1.448/0.0035 = 413$ deg/s.

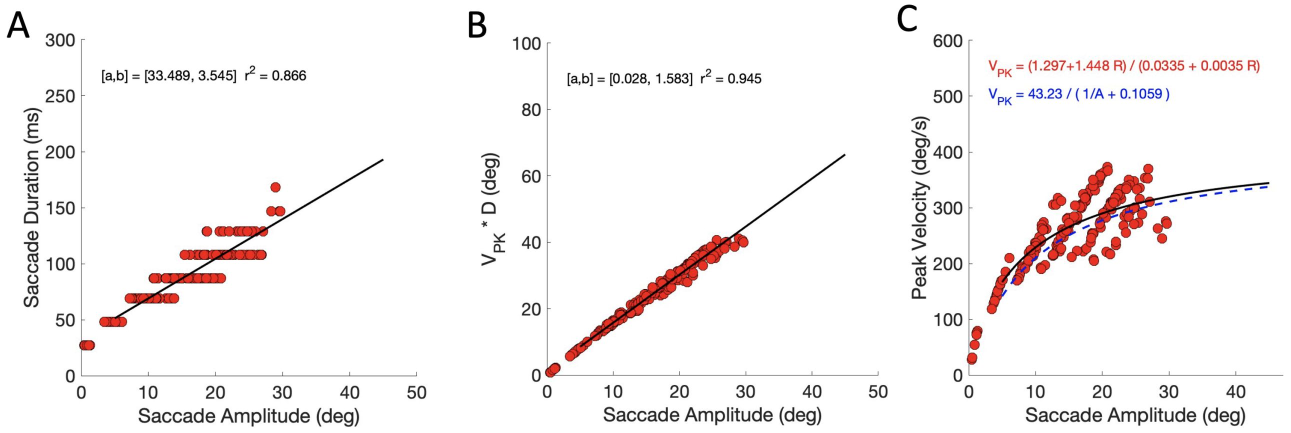

Figure S3. *Main-sequence behaviors for the 199 saccades from the zero-initial paradigm.*

3.3 Straight trajectories

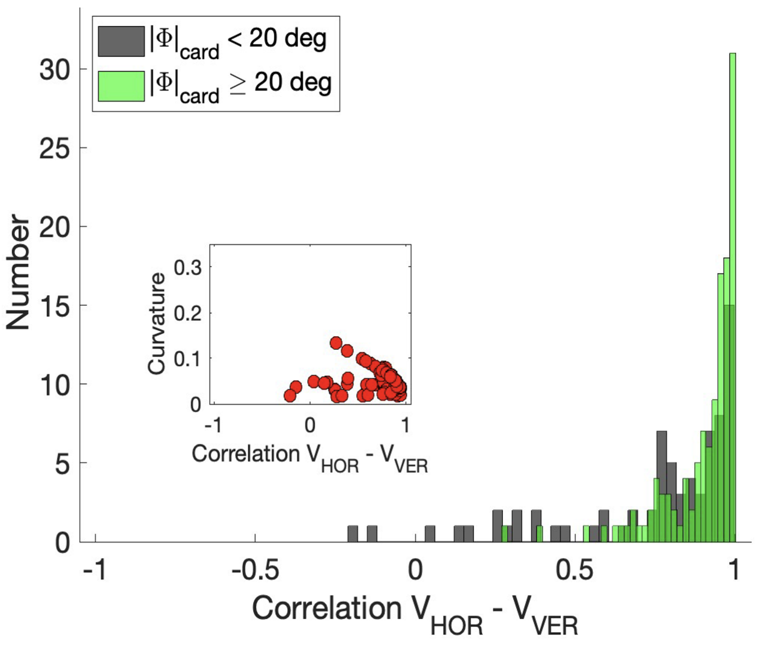

Figure S4. *Correlation histograms between the horizontal and vertical velocity profiles of oblique saccades (green; more than 20 deg away from the cardinal axes) and near-cardinal saccades (gray). Inset: the relation between curvature and the velocity-profile correlations is not significant.*

In Figure S4 it is shown that the horizontal and vertical velocity profiles of the oblique saccades of the zero-initial paradigm (with their vector directions at least 20 deg away from the cardinal axes) correlate well, with most correlation coefficients close to 1.0 and all curvatures smaller than 0.15 (inset; see also Fig. 8C of the manuscript).

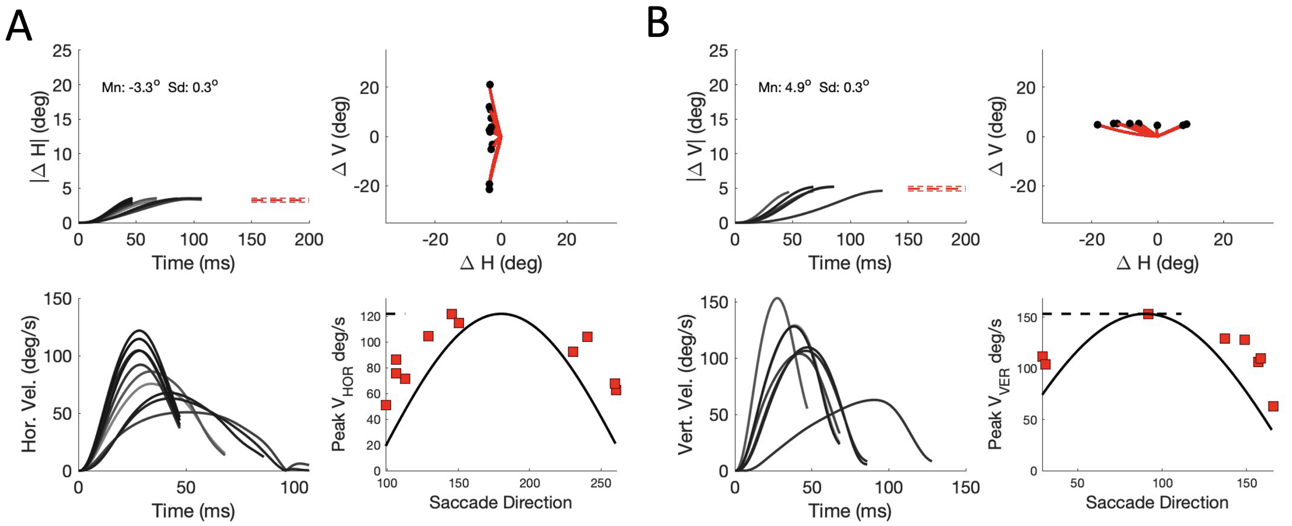

Figure S5. *Component stretching in oblique saccades. **(A)** Fixed horizontal component, **(B)** Fixed vertical component.*

Figure S5 illustrates how the peak velocity of a given fixed horizontal (a) (mean ΔH = -3.3 deg) or vertical (b) (mean ΔV = +4.9 deg) component depends on the size of the orthogonal component, and how this relates to the saccade direction. The lower-left panels show that the velocity profiles vary considerably for the different selected responses, indicating that the independent model is unable to explain the behavior of the system. The solid curves in the lower-right panels show the predictions of the common-source model under the simplifying assumption that the vectorial amplitude-peak velocity relation is direction independent (see, however, Fig. 11 of the manuscript).

3.4 Muscle controls

Figure S6 shows the muscle-control signals for the saccades elicited by the zero-initial paradigm. Figure S6A provides the details of a single example saccade to the right and upward, and clearly demonstrates the grouping of the six muscles into agonists and antagonists. It also shows the pulse-step nature of the three agonists (LR, SR and IO) and the reverse behavior for the antagonists (MR, IR and SO). Figure S6B shows the LR/MR responses for near-horizontal saccades, whereas Fig. S6C provides the data of IO/SO/IR/SR for near-vertical saccades.

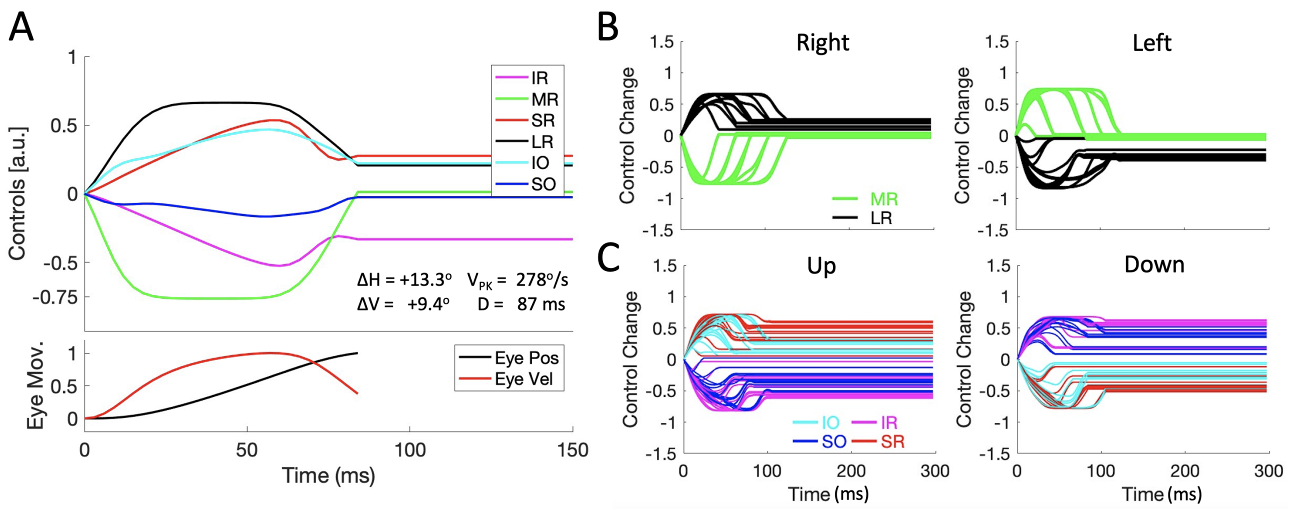

Figure S6. *Muscle control signals for the zero-initial paradigm.* ***(A)*** *Controls for a right- and upward saccade with* $[R, \Phi]$ *= [16.3, 35.3] deg. Top: control signals of the six muscles. Bottom: vectorial position (black) and velocity (red).* ***(B)*** *LR and MR control signals for near-horizontal saccades.* ***(C)*** *SR,IR, SO and IO controls for near-vertical saccades. Note that SR/IO and SO/IR function as agonists.*

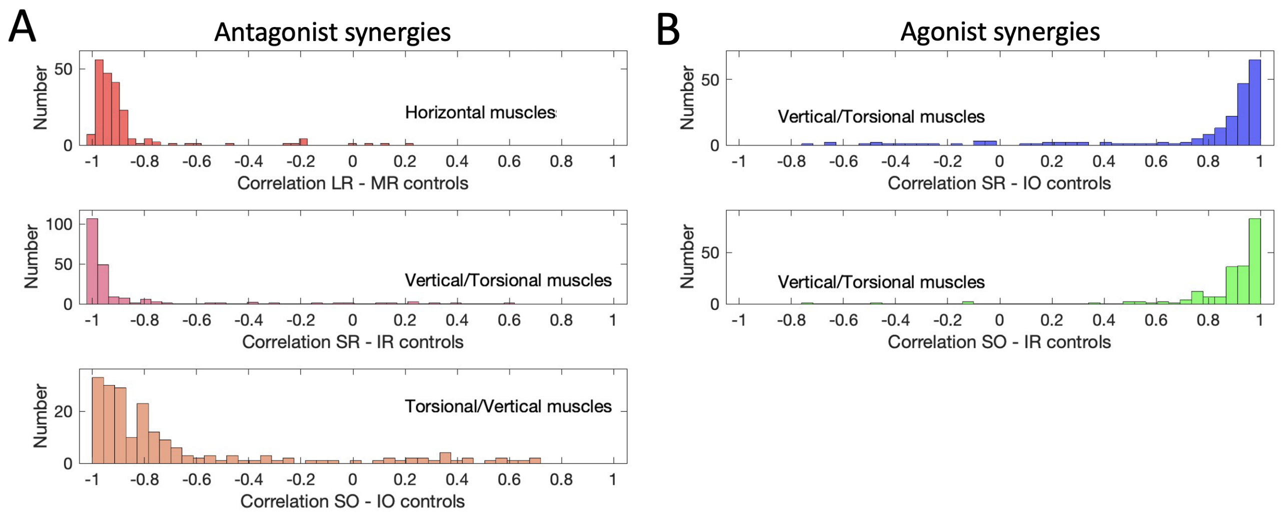

Figure S7. *Muscle synergies for all saccades in the zero-initial paradigm, grouped as* ***(A)*** *antagonists and* ***(B)*** *agonists.*

Figure S7 shows the correlations between antagonist pairs and agonist pairs for the zero-initial saccades. The correlations for the agonists cluster near the optimal value of +1.0, whereas the LR/MR and SR/IR antagonists cluster near the optimal value of -1.0. Note that the SO/IO antagonistic behavior is more variable, which is due to Listing's Law, allowing the eye only a very small range of cyclotorsion.

3.5 Direct stimulation of the SO muscle.

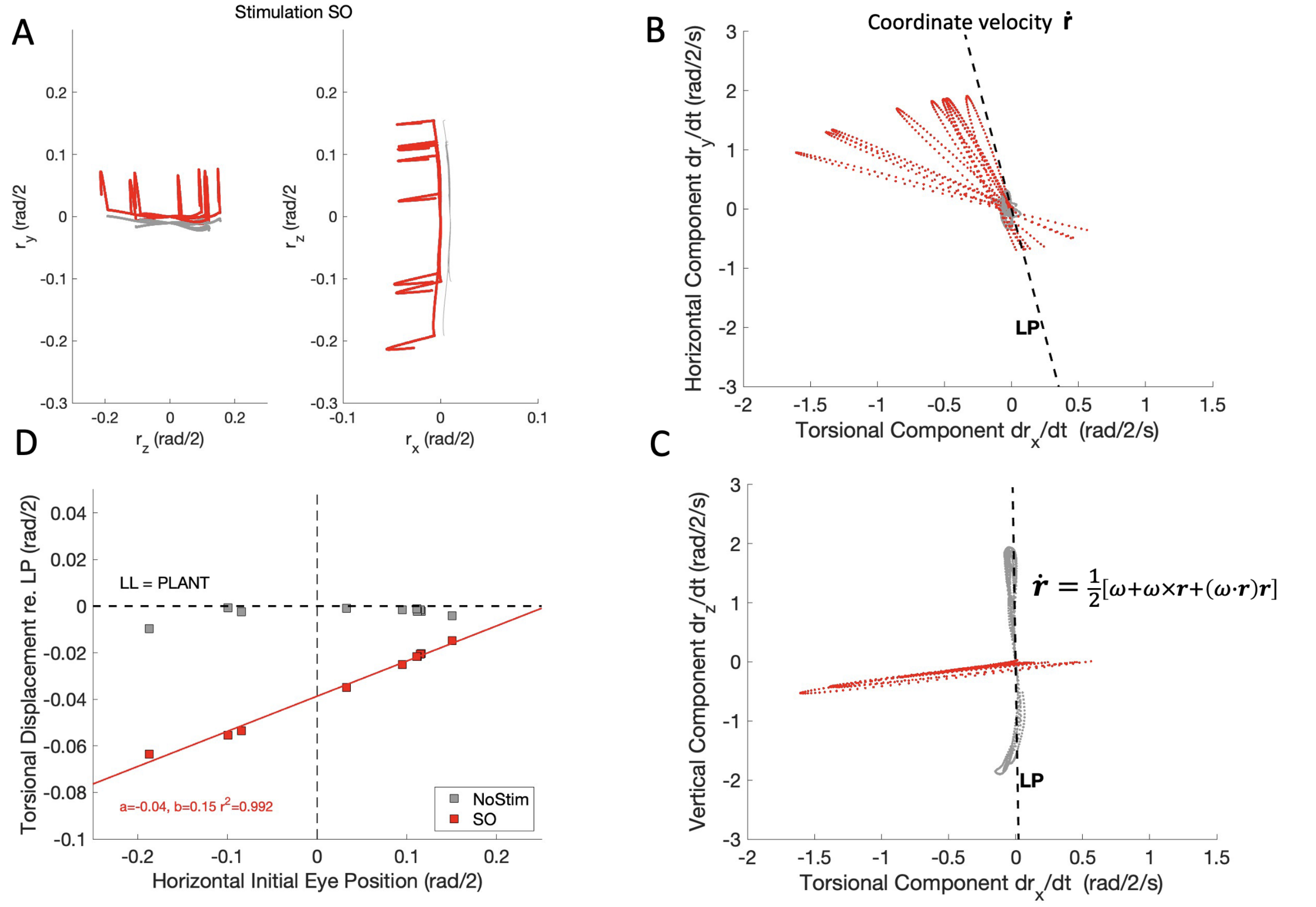

Figure S8. *(A) Eye-movement trajectories with respect to Listing's Plane (LP) after stimulating the SO muscle with a Gaussian pulse of 21 ms width from different initial horizontal eye orientations. The control data without stimulation (grey) have been shifted by 0.01 rads/2 for visual purposes.* ***(B,C)*** *The stimulation yields a vertical downward movement with a counterclockwise torsional component, whereby the size of the latter varies linearly with the initial horizontal position* ***(D)****.*

Figure S8 describes the resulting saccade trajectories when stimulating the SO muscle after a horizontal saccade. Figure S8A depicts the experimental paradigm: an eye movement is made from straight ahead to a horizontal target location, after which the SO muscle (red traces) is stimulated by a Gaussian pulse with a width of 21 ms (the same pulse as in Fig. 15 of the paper). The stimulation elicits a downward (top-left) and in-torsional eye movement from 10 different positions (cf. Hepp et al. (1989)). The right-hand panels show an (r_x, r_y) view of the traces in the laboratory frame (cf. with Fig. S2B). The vertical-torsional movements elicited by the stimulation produced a coordinate velocity, $\dot{\mathbf{r}}$, that clearly deviated from Listing's plane, as shown in Figure S8B,C. The light-grey traces are the horizontal eye movements before the stimulation, and they define Listing's Plane (dashed black lines, LP). Figure S8D shows that the cumulative deviation from

LP, Δr_x, depends on the initial horizontal eye orientation. Note that the obtained relationship is remarkably linear:

$$\Delta r_x^{SO}(r_z) = -0.04 + 0.15 \cdot r_z \quad (r^2 = 0.992)$$

3.6 Half-angle behavior

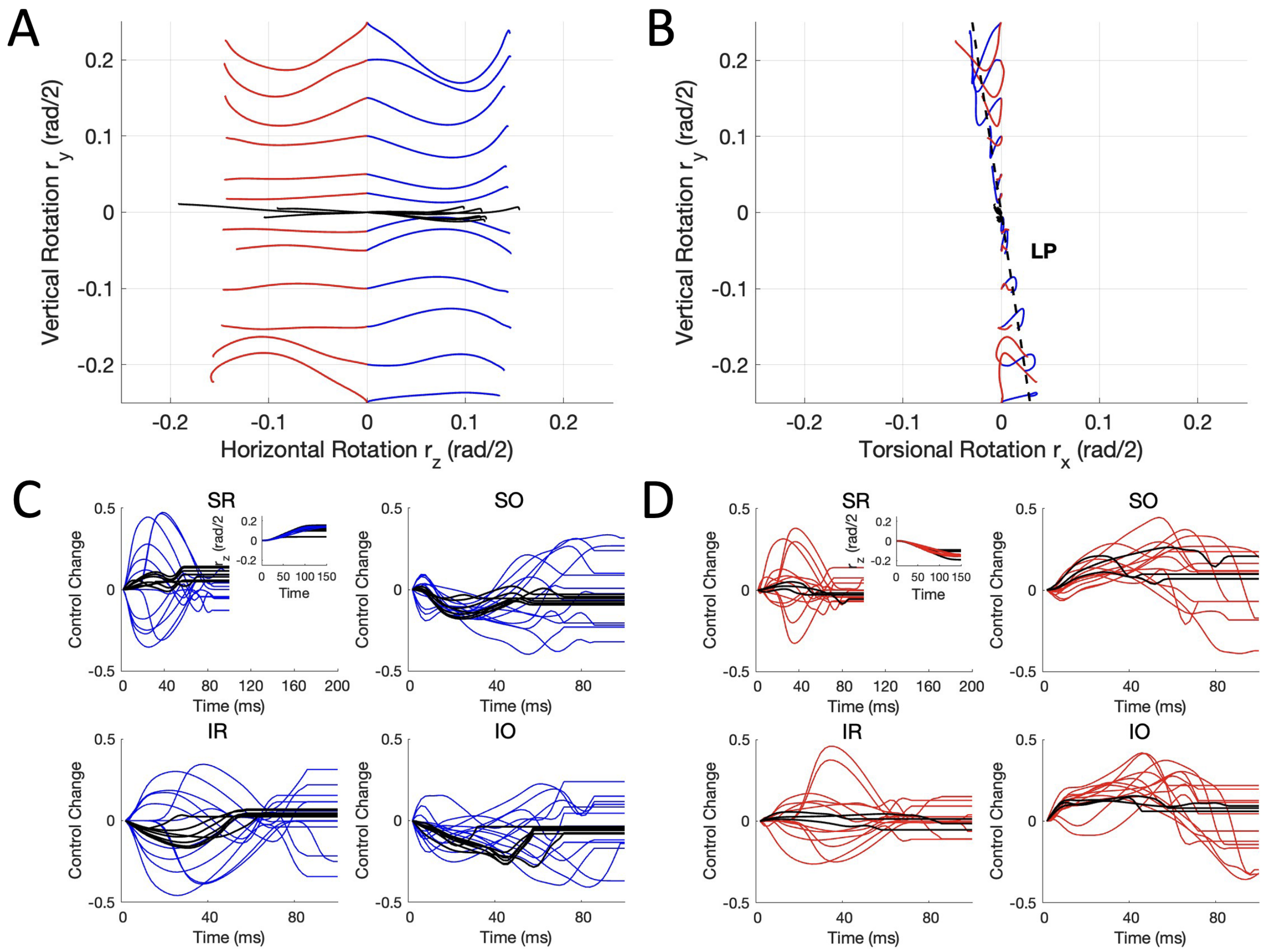

Figure S9. **(A,B)** *Horizontal left (blue)/right (red) eye-movement trajectories of 15 deg amplitude elicited from different vertical initial eye orientations. The black traces are the horizontal saccades from the zero-initial paradigm. (A) zy plane. (B) xy plane. Dashed line, LP = Listing's Plane.* **(C,D)** *Net muscle controls for the four vertical-torsional muscles during the horizontal saccades:* ***(C)*** *Left (trajectories in inset upper-left);* ***(D)*** *Right (trajectories in inset upper-left). Note the substantially larger range for the vertical-torsional pulse signals, especially for the SR/IR muscles, when compared to the saccades starting at (0,0,0) (black).*

In Figures S9 and S10 we illustrate how the vertical-torsional muscles are recruited for horizontal saccades that start at different vertical initial eye orientations. These saccades were generated by a separate continuous paradigm, in which the first saccade brought the eye from (0,0,0) to (0,-0.25,0), the second was the first rightward saccade to (0, -0.25, -0.15), the third an oblique one to (0,-0.20, 0), etc., working its way up to (0, +0.25, -0.15). Then the reverse sequence continued with leftward saccades, from (0.0.25,0.15) down to (0,-0.25,0.15). Figures S9A,B show the resulting 24 left/right saccade trajectories of approximately equal amplitude from the 12 vertical eye orientations between -28 and +28 deg, as well as the horizontal

saccades taken from the zero-initial paradigm (black traces). In Fig. S9C,D the saccadic pulse activities of the four vertical/torsional muscles are shown for the leftward (C) and rightward (D) saccades from both paradigms. Clearly, the pulses for the saccades from different initial vertical positions span a much larger range than the vertical/torsional pulses for the zero-initial saccades (black).

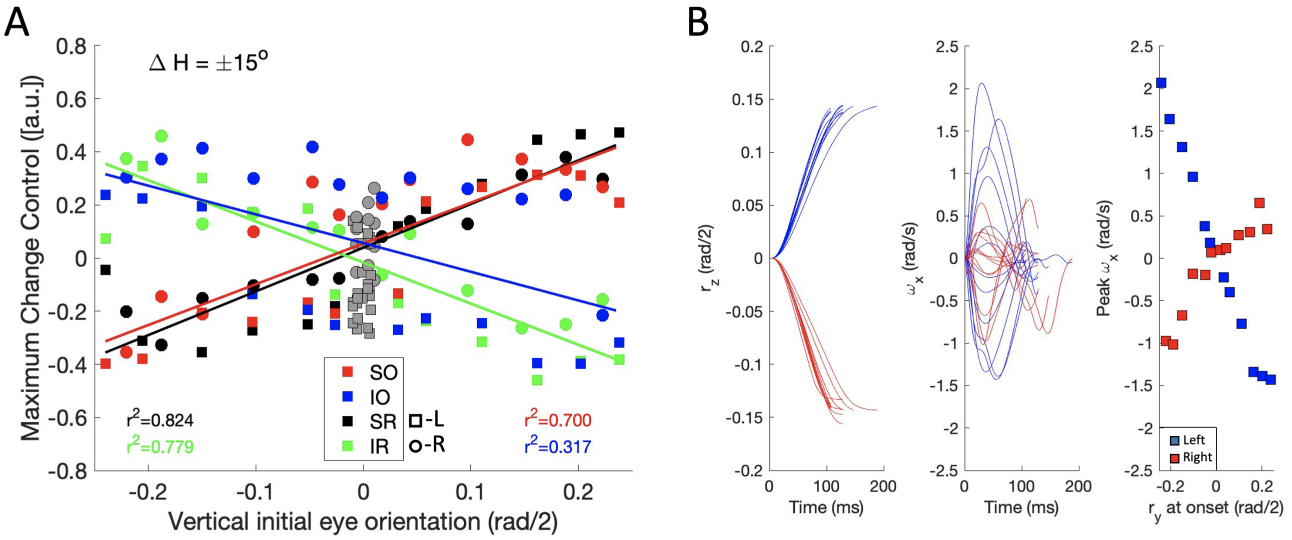

Figure S10. **(A)** *Signed pulse-peak amplitude of the net control signal of the four vertical-torsional muscles (legend) as function of the initial vertical eye orientation. Same data as in Fig. S9. Grey symbols: the horizontal zero-initial saccades. Three of the muscles are systematically, and nearly linearly, recruited as function of* r_y^{on}. *Despite the considerable trial-to-trial variability, the* r^2 *values for the linear fits are high, except for the IO muscle, the activity of which better reflects differences in right- (circles) vs. leftward (squares) saccades.* ***(B)*** *The torsional component of the resulting angular velocity vector for these horizontal saccades varies (nearly) linearly with the initial vertical eye orientation according to the half-angle rule.*

Figure S10A quantifies the qualitative observations of Fig. S9 by plotting the (signed) maximum pulse activity of the four muscles as a function of the initial eye orientation. Each muscle is identified by a different color. The zero-initial saccades are identified by the gray symbols. Squares denote leftward saccades, circles are rightward. Note that the pulses for three of the four muscles (SR, IR and SO) show a strong linear relationship with vertical initial eye orientation ($r^2 > 0.7$), with the IO muscle showing more variability, mainly related to left- vs. rightward saccades. The linear regression lines for the four muscles are parameterized by:

$$\begin{aligned}
P_{SR}(r_y) &= +0.039 + 1.645 \cdot r_y \quad (r = 0.91) \\
P_{IR}(r_y) &= -0.017 - 1.548 \cdot r_y \quad (r = 0.88) \\
P_{SO}(r_y) &= +0.055 + 1.536 \cdot r_y \quad (r = 0.84) \\
P_{IO}(r_y) &= +0.057 - 1.080 \cdot r_y \quad (r = 0.56)
\end{aligned}$$

These four relations nicely show the antagonistic nature of the vertical-torsional system. These results also indicate that the half-angle rule (at least a correlate of the rule) is encoded in large part by the motor control. This half-angle behavior is illustrated for the horizontal saccades in Fig. S10B, which shows that the peak

torsional component of the angular velocity axis for the leftward and rightward saccades is well described by linear regressions:

$$\begin{aligned}\omega^x_{PK}(\Delta H > 0) &= +0.099 - 7.626 \cdot r_y \quad (r^2 = 0.99)\\ \omega^x_{PK}(\Delta H < 0) &= -0.095 + 3.416 \cdot r_y \quad (r^2 = 0.88)\end{aligned}$$

The relationship between the muscle-activation patterns and the resulting half-angle behavior, however, results to be distributed across the four muscles in a non-trivial way because of the nonlinear transformations between motor angles and resulting eye orientation (S1-S5).

REFERENCES

Hepp, K., Henn, V., Vilis, T., and Cohen, B. (1989). *The Neurobiology of Saccadic Eye Movements. Chapter 4: "Brainstem regions related to saccade generation"*, vol. IV, pp. 105-212 (Elsevier)

Javanmard Alitappeh, R., John, A., Dias, B., Van Opstal, A. J., and Bernardino, A. (2023). Emergence of human oculomotor behavior in a cable-driven biomimetic robotic eye using optimal control. *IEEE Transactions on Cognitive and Developmental Systems* , in press

John, A., Van Opstal, A. J., and Bernardino, A. (2023). A cable-driven robotic eye for understanding eye-movement control. *IEEE Xplore, ICARA-2023* , 128–133.doi:10.1109/ICARA56516.2023.10126021

Miller, J. M. and Robinson, D. A. (1984). A model of the mechanics of binocular alignment. *Comp. Biomed. Res.* 17, 436–470. doi:https://doi.org/10.1016/0010-4809(84)90012-0

Shadmehr, R., De Xivry, J. J. O., Xu-Wilson, M., and Shih, T.-Y. (2010). Temporal discounting of reward and the cost of time in motor control. *Journal of Neuroscience* 30, 10507–10516

Shadmehr, R. and Mussa-Ivaldi, S. (2012). *Biological learning and control: how the brain builds representations, predicts events, and makes decisions (Chapter 11)* (MIT Press)
